# *In silico* molecular studies of Phosphinogold(I) thiocarbohydrate complexes: insights into multi-target anticancer mechanisms

**DOI:** 10.3389/fchem.2025.1533026

**Published:** 2025-06-12

**Authors:** Alkhair Adam Khalil Mohamed, Isaac Asiamah, Ghazi Elamin, James Darkwa, Christian K. Adokoh

**Affiliations:** ^1^ Department of Biomedical Sciences, School of Allied Health Sciences, College of Health and Allied Sciences, University of Cape Coast, Cape Coast, Ghana; ^2^ Department of Chemistry, School of Physical Sciences, College of Agriculture and Natural Sciences, University of Cape Coast, Cape Coast, Ghana; ^3^ Department of Pharmaceutical Chemistry, College of Pharmacy, Karary University, Khartoum, Sudan; ^4^ Department of Chemical Sciences, Faculty of Science, University of Johannesburg, Johannesburg, South Africa; ^5^ Department of Forensic Science, School of Biological Sciences, College of Agriculture and Natural Sciences University of Cape Coast, Cape Coast, Ghana

**Keywords:** anticancer activity, cytotoxicity, MM-GBSA, molecular docking, structure activity relationship, thiocarbohydrate Phosphinogold(I) complexes

## Abstract

**Introduction:**

This study employed in silico methods to investigate the anticancer potential and mechanisms of twenty novel phosphinogold(I) thiocarbohydrate complexes.

**Methods:**

Molecular docking and Prime MM-GBSA screening of seventeen cancer-related protein targets, including Human Double Minute 2 protein (HDM2), DNA methyltransferase-1 (DNMT1), Protein Kinase B (AKT2), and Poly (ADP-ribose) polymerase 1 (PARP-1), were conducted. Molecular dynamics simulations were performed for complex **9**.

**Results:**

Virtual screening revealed strong binding affinities for several complexes, often surpassing native ligands. All the complexes except **16**, **18**, and **19** exhibited strong binding affinity with one or two cancer protein targets compared to native ligands. Complex **9** emerged as the best candidate, demonstrating promising binding affinity particularly against AKT2 (–82.40 kcal/mol) and PARP-1 (–75.7 kcal/mol). Molecular dynamics simulations of complex **9** with PARP-1 and AKT2 revealed distinct binding profiles, with a more stable interaction with PARP-1, suggesting its potential for disrupting DNA repair mechanisms. Binuclear complexes generally exhibited higher affinities than mononuclear counterparts, particularly for DNMT1 and HDM2. Complex **13** demonstrated high *in vitro* activity against prostate, colon, and breast cancer cell lines (IC50 = 0.03, 0.25, and 0.07 μM respectively), collaborating with a significant interaction with Human Epidermal Growth Factor Receptor 2 (HER2) (–71.15 kcal/mol binding affinity) *in silico*. While acetylation decreased binding affinity; it enhanced cellular activity as reported in *in vitro* studies indicative of the need to balance lipophilicity and binding strength in future ligand design.

**Discussion:**

These findings provide valuable insights into multi-target anticancer mechanisms, with a particular emphasis on complex **9** as a potential PARP-1 inhibitor, and guide future optimization and experimental validation of these novel gold-based complexes. The stable interaction of complex **9** with PARP-1 highlights PARP-1 as a particularly promising therapeutic target. Binuclear complexes' superior affinities for DNMT1 and HDM2 suggest structural advantages for multi-target inhibition.

**Conclusion:**

The paradoxical effect of acetylation underscores the importance of balancing lipophilicity and binding strength in ligand design.

## Introduction

The exploration of gold-based compounds for therapeutic applications has a rich history, dating back to ancient civilizations where gold was utilized for treating various ailments. Recent advancements have reignited interest in these compounds, particularly due to their potential anticancer properties. However, the clinical application of gold complexes is often limited by the toxicity of their ligands and their biocompatibility issues (Biebuyck et al., 1994).

In a previous study, we synthesized and characterized novel phosphinogold(I) thiocarbohydrate complexes, which were designed to overcome the limitations associated with traditional gold complexes (Adokoh et al., 2017). The choice of targeting these phosphinogold(I) thiocarbohydrate complexes is as result of their promising cancer treatment due to their potent cytotoxicity, ability to induce apoptosis through mitochondrial pathways, the tunable nature of their phosphine ligands to enhance anticancer activity and selective targeting characteristics of thiocarbohydrate ligands. By combining gold(I) with phosphine and thiocarbohydrate ligands, the complex can leverage multiple mechanisms: enzyme inhibition, oxidative stress induction, and selective targeting of cancer cells, thus, a potential for synergistic effects is expected (Zarewa et al., 2023; Keter et al., 2014). These new compounds were synthesized through the reaction of n-gluconamidoalkyl thiols with various gold precursors, resulting in a series of complexes that exhibit promising anticancer activities against different cancer cell lines, including breast and prostate cancer (Adokoh et al., 2017). The anticancer evaluation of these complexes revealed that certain dinuclear complexes demonstrate significantly higher tumor selectivity and activity than their mononuclear counterparts. One such complex exhibited remarkable tumor selectivity (TS) value of approximately 24, indicating its potential as a targeted therapeutic agent (Adokoh et al., 2017). Furthermore, *in vitro,* studies highlight the crucial role of the length of the alkyl chains in anticancer efficacy, with longer chains generally correlating with improved selectivity and activity (Keter et al., 2014).

Despite these promising results, the precise mechanism of action of these phosphinogold(I) complexes remains to be elucidated. Therefore, the present *in silico* investigation is expected to provide insights into how these complexes interact at the molecular level with cancer cells. This computational approach is designed to help identify potential targets and pathways involved in the anticancer activities reported for these complexes to pave the way for future experimental validation and optimization of these novel compounds. The findings from this investigation could significantly contribute to the development of safer and more effective gold-based anticancer therapies.

The *in silico* analysis of phosphinogold(I) thiocarbohydrate complexes can provide insights into their potential mechanisms of action as anticancer agents. We hope to verify several hypotheses based on computational modeling and molecular docking studies. Firstly, we hypothesized that the complexes may bind effectively to specific target proteins involved in cancer cell signaling pathways. *In silico* docking studies could reveal high-affinity interactions with proteins such as kinases or transcription factors that regulate cell proliferation and survival, indicating a potential mechanism for inhibiting tumor growth (Steven, 2003; Yip and Papa, 2021). Secondly, the computational analysis may suggest that the phosphinogold(I) complexes exhibit higher binding affinities for cancer cell-specific targets compared to normal cell targets. This selectivity could be attributed to the unique structural features of the complexes, such as the presence of thiocarbohydrate ligands, which may enhance their interaction with tumor-specific receptors or enzymes (Sankarganesh et al., 2019). Also, it is possible that the binding of these complexes to target proteins induces conformational changes that disrupt normal protein function. *In silico* simulations could help to visualize these changes, providing evidence that the complexes interfere with the activity of critical proteins involved in cancer cell survival and proliferation (Bajracharya et al., 2022). Additionally, the complexes may be hypothesized to generate reactive oxygen species through their interactions with cellular components. *In silico* studies could model the redox potential of the complexes, suggesting that they may promote oxidative stress in cancer cells, leading to cell death (Arojojoye et al., 2022; Bhattacharjee et al., 2022; Nath et al., 2023; Ndagi et al., 2017; Selivanov et al., 2011; Yu et al., 2022). Again, computational studies might indicate that these complexes can bind to DNA repair proteins, inhibiting their function. This could lead to an accumulation of DNA damage in cancer cells, ultimately resulting in cell cycle arrest and apoptosis. *In silico* analysis could identify potential binding sites on these proteins (Kim et al., 2021; Kim et al., 2019).

Successfully verifying these hypotheses can guide future experimental studies to validate the proposed mechanisms of action for phosphinogold(I) thiocarbohydrate complexes as anticancer agents. By leveraging *in silico* approaches, we hope to gain a deeper understanding of the molecular interactions and pathways involved in the anticancer activity of these complexes.

## Methods

### 3D modeling and preparation of the Phosphinogold(I) thiocarbohydrate complexes

**FIGURE 1:**
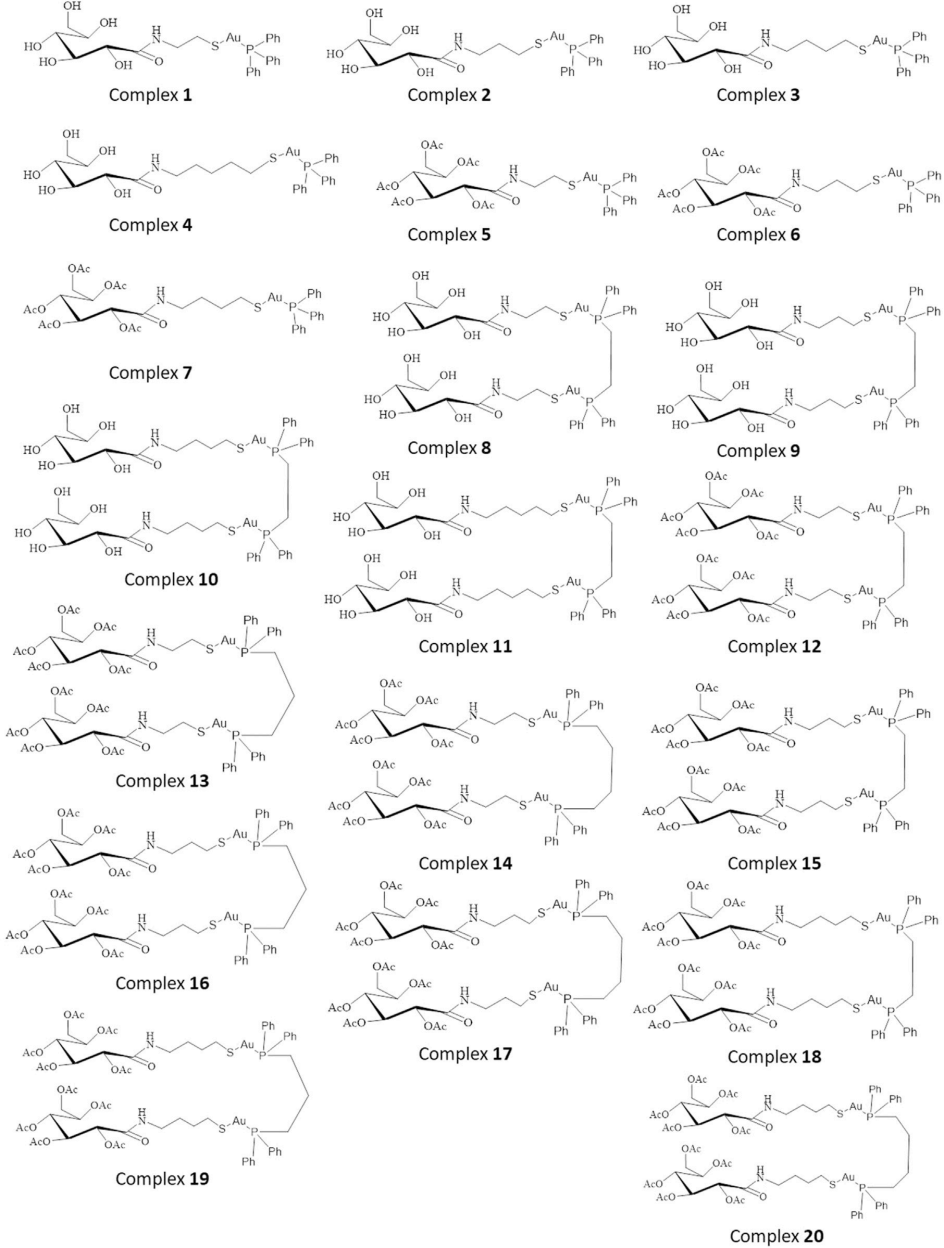
Two-dimensional structures of 20 Phosphinogold(I) Thiocarbohydrate Complexes.

The complexes were first sketched using the 2D sketcher of Maestro (Schrödinger), by drawing the triphenylphosphine, bisdiphenylphosphines, and seven ligands as building blocks for the final assembly of the mono and binuclear Phosphinogold(I) thiocarbohydrate complexes as illustrated in Figure 1. The sketched molecules in Figure 1 were then assembled as the 20 mono and binuclear complexes described by (Adokoh et al., 2017) utilizing the single complex builder within Maestro (Schrödinger, 2023a). This was done by selecting gold(I) as the central atom(s), and linear geometry for the final complex around the central atom (Au). The complex builder was run to join the gold atom(s) to the phosphine’s phosphorus atom(s) at one end, and to the sulfur atom(s) of the thiocarbohydrate ligand(s) at the opposite end. The final assembled complexes are illustrated in Figure 1. The LigPrep tool in (Schrödinger, 2023b) was used to convert the 2D structures into 3D structures. Subsequently, each complex’s geometry was optimized using OPLS4 force field minimization by running the complex cleanup tool in Maestro. The final library of prepared complexes was then used to perform the subsequent molecular docking and MM-GBSA free energy calculation studies.

### Protein targets selection and preparation

The 20 complexes have previously shown remarkable anticancer activities in 3 cell lines of the breast (MCF7), prostate (PC3), and colon (HCT116) cancers (Adokoh et al., 2017). However, the mechanism of action of these complexes is not yet known. To investigate the possible mechanisms for the observed anticancer activities, a thorough literature review was initiated to look for the available known targets involved in the three types of cancer cells that have significant effects on their life cycle. The review discovered 17 targets that could be investigated through an *in silico* approach to evaluate their binding interactions and to estimate binding affinities toward the Phosphinogold(I) Thiocarbohydrate Complexes, which will make it possible to hypothesize possible mechanisms for the observed anticancer activities. Table 1 summarizes the selected targets and their involvement in various types of cancer. The crystallized structures of the targets were downloaded as pdb files from the protein data bank, accessible at https://www.rcsb.org/, then they were loaded into the protein preparation workflow in Maestro (Schrödinger, 2023c) at the default settings. Protein preparation for molecular docking simulations was conducted through a series of sequential steps, involving filling in missing side chains, assigning bond orders, placing hydrogens, generating het states at pH 7.4 ± 2, deleting bulk waters, optimizing hydrogen-bond assignments at pH 7.4, and lastly, energy minimization of the protein using the OPLS4 force field. The co-crystallized ligands or inhibitors (Table 1) from the protein targets were extracted, prepared in LigPrep (Schrödinger, 2023d), and subsequently used to validate docking protocols and they were used as reference to compare binding affinities across the 20 phosphinogold(I) thiocarbohydrate complexes using Glide (Schrödinger). It is important to mention that, the target protein β-catenin with PDB ID: 1JDH had no cocrystallized ligand or inhibitor, therefore, the binding site was determined utilizing the SiteMap function within Maestro software, and for subsequent docking studies, PRI-724 (also known as Foscenvivint); a known potent inhibitor for β-catenin signaling pathway (Schmidtova et al., 2021), was selected as the standard ligand for 1JDH to compare binding affinity between ligands to this target.

**TABLE 1:** Protein targets used for virtual screening of the gold(I) complexes.

Target PDB ID	Cocrystallized ligand/inhibitor	Target description	Type of cancer involved in	References
1HCK	ATP	Human cyclin-dependent kinase 2	Breast	Schulze-Gahmen et al. (1996)
1JDH	PRI-724	β-catenin and HTCF-4	Colon	Graham et al. (2001)
1JFF	Taxol	Alpha-Beta-tubulin dimer	Breast	Löwe et al. (2001)
1M17	Erlotinib	Epidermal Growth Factor Receptor (EGFR) tyrosine kinase domain	Breast	Stamos et al. (2002)
1O6L	Phospho amino phosphonic acid-adenylate ester	Activated Akt/protein kinase B	Prostate	Yang et al. (2002)
2GU8	CHEMBL213618	Akt/protein kinase B	Prostate	Lin et al. (2006)
3D0E	GSK690693	Human AKT2	Prostate	Heerding et al. (2008)
3L3M	A927929	Poly (ADP-ribose) polymerase (PARP-1)	Breast/Prostate	Penning et al. (2010)
3PP0	CHEMBL1614726	Kinase domain of human epidermal growth factor receptor 2 (HER2)	Breast	Ishikawa et al. (2011)
3RCD	TAK-285	Kinase domain of human epidermal growth factor receptor 2 (HER2)	Breast	Ishikawa et al. (2011)
4JT5	TORKinib	Mammalian target of rapamycin (mTOR)	Breast	Yang et al. (2013)
4OBE	GDP	GDP-bound Human KRas	Colon	Hunter et al. (2014)
4WXX	S-adenosyl-l-homocysteine	DNA methyltransferase_1	Colon	Zhang Z.-M. et al. (2015)
4XV2	Dabrafenib	B-Raf Kinase	Colon	Zhang C. et al. (2015)
5HMH	CHEMBL3805372	Human Double Minute 2 protein (HDM2)	Prostate	Bogen et al. (2016)
8HOI	Sonrotoclax	B-cell lymphoma 2 (BCL2)	Breast	Liu et al. (2024)
8Q61	PubChem CID: 163231351	Human Akt2	Breast/Prostate	Page et al. (2022)

### Molecular docking

Molecular docking was performed using Glide (Schrödinger, 2023a) software. The procedure was initiated by selecting each prepared target protein as the macromolecule and the 20 prepared gold(I) complexes as the ligands. A receptor grid box using glide was previously generated, centered on the position of the cocrystallized ligand, and a midpoint box of 10 Å diameter in all three coordinates. Flexible ligand sampling with extra precision (XP) was selected to run the molecular docking simulations. The output settings of the docking results were kept at their default values to report the best pose with the highest docking score for each ligand.

### Validation of docking protocol

**FIGURE 2:**
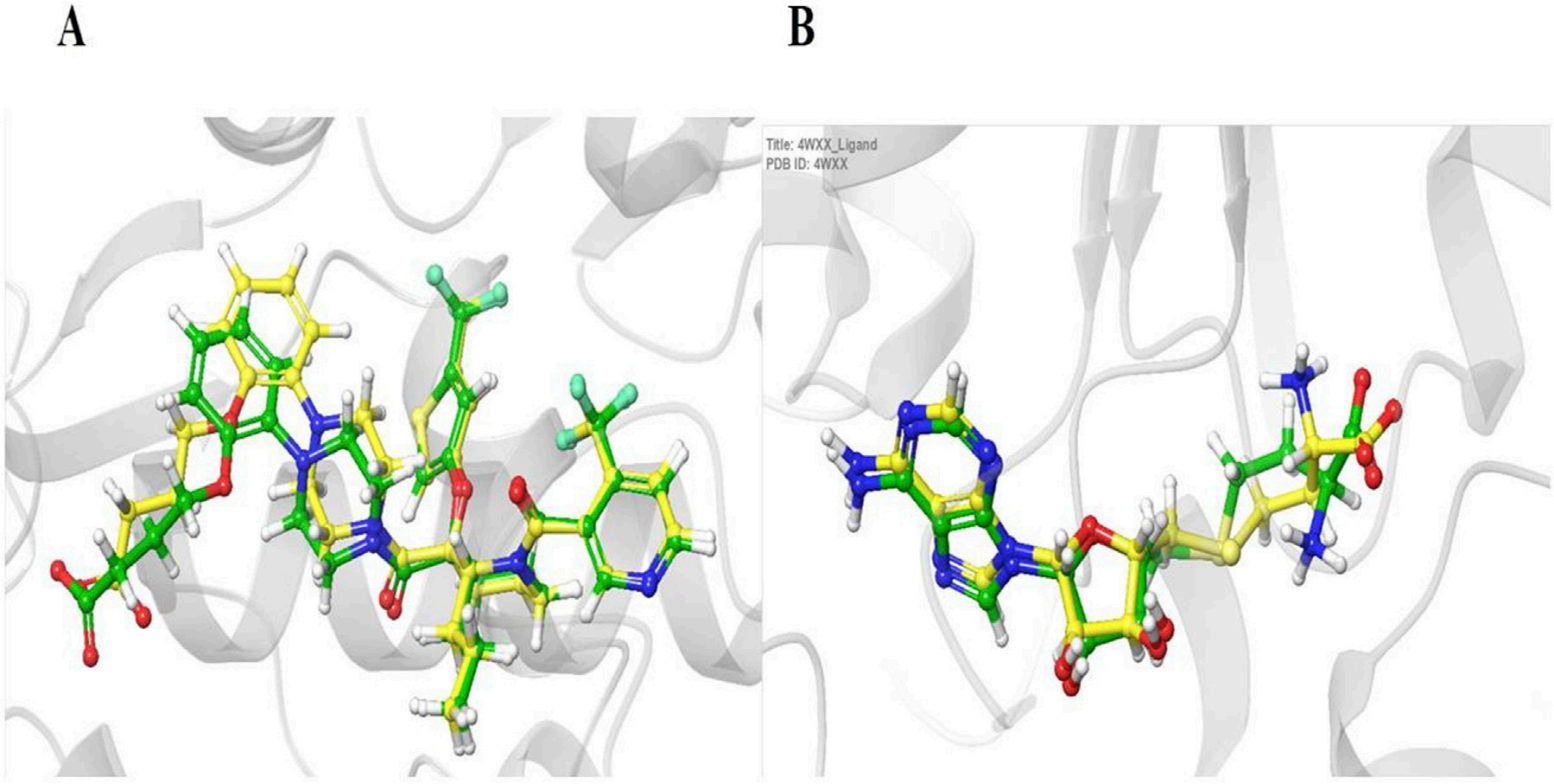
Validation of the docking protocol using redocking. Panels **(A**, **B)** show the superimposition of the experimentally determined (cocrystallized) ligand (yellow) and the computationally redocked ligand (green) for two target proteins: HDM2 (PDB ID: 5HMH, A) and DNAMT1 (PDB ID: 4WXX, B). The low root mean square deviation (RMSD) values of 0.96 Å **(A)** and 0.95 Å **(B)** demonstrate the reliability of the docking methodology in accurately reproducing known ligand binding poses.

The reliance on redocking in validating docking protocols (Table 2) and assessing accuracy will help assess benchmarking performance. When there is a high level of repetition between the experimentally determined and docked poses, it gives confidence that the docking results reflect, to a high degree, what is happening. In the present study, the native ligands were docked using the same grid box generated for ligand docking, and the poses of the co-crystallized and docked ligands were compared by calculating root mean square deviation (RMSD) (Figure 2). Following this outcome, if the RMSD is small (preferably less than 2.0), then the docking protocol is considered valid (Hevener et al., 2009; Jain, 2008).

**TABLE 2:** Docking scores and binding free energy of target’s cocrystallized ligands or inhibitors.

Target PDB ID	Cocrystallized ligand/inhibitor	Docking score kcal/mol	RMSD	MM-GBSA Kcal/mol	Target description
1HCK	ATP	−18.36	2.03	−8.7	Human cyclin-dependent kinase 2
1JDH	PRI-724	−3.65	-	−28.6	β-catenin and HTCF-4
1JFF	Taxol	−7.54	0.70	−77.7	Alpha-Beta-tubulin dimer
1M17	Erlotinib	−9.52	1.56	−64.4	Epidermal Growth Factor Receptor (EGFR) tyrosine kinase domain
1O6L	Phospho amino phosphonic acid-adenylate ester	−17.11	1.42	−39.5	Activated Akt/protein kinase B
2GU8	CHEMBL213618	−13.39	0.53	−72.1	Akt/protein kinase B
3D0E	GSK690693	−8.29	0.69	−94.8	Human AKT2
3L3M	A927929	−9.11	1.09	−69.3	Poly (ADP-ribose) polymerase (PARP-1)
3PP0	CHEMBL1614726	−14.55	2.02	−91.8	Kinase domain of human epidermal growth factor receptor 2 (HER2)
3RCD	TAK-285	−9.86	1.64	−70.0	Kinase domain of human epidermal growth factor receptor 2 (HER2)
4JT5	TORKinib	−10.20	0.00	−59.9	Mammalian target of rapamycin (mTOR)
4OBE	GDP	−15.98	0.96	−40.8	GDP-bound Human KRas
4WXX	S-adenosyl-l-homocysteine	−8.06	0.95	−68.6	DNA methyltransferase_1
4XV2	Dabrafenib	−11.52	0.20	−69.4	B-Raf Kinase
5HMH	CHEMBL3805372	−13.71	0.96	−94.4	Human Double Minute 2 protein (HDM2)
8HOI	Sonrotoclax	−10.56	0.70	−104.6	B-cell lymphoma 2 (BCL2)
8Q61	PubChem CID: 163231351	−9.33	0.63	−59.3	Human Akt2

### Prime MM-GBSA calculations

The MM-GBSA method combines molecular mechanics force fields (MM) with implicit solvation models (Generalized Born and Surface Area, (GBSA)) to estimate the binding free energy (binding affinity). MM-GBSA is computationally efficient and often used as a first-pass estimate of binding affinity. Prime MM-GBSA can be performed without running a full-fledged MD simulation, utilizing the docked poses as a starting point.

Molecular docking relies on scoring functions to assess the quality of the predicted binding poses generated during conformational search. These functions utilize approximations to streamline the calculations, enabling high-throughput screening of potential drug candidates. Due to the use of approximate scoring functions, molecular docking often falls short of accurately predicting binding energies when compared to experimental measurements. Although, numerous docking programs effectively identify potential ligand binding conformations, a universal scoring function that accurately predicts binding energies for all molecules and protein families remains elusive. Consequently, rescoring steps after molecular docking are often essential to refine the initial predictions (Zhang et al., 2017). On the other hand, MM-GBSA utilizes molecular mechanics and free energy calculations to consider entropic contributions and thus provide more accurate binding affinity predictions (Sgobba et al., 2012). MM-GBSA is computationally more expensive but offers greater accuracy and insights into binding mechanisms, making it ideal for ranking and refining promising ligands (Genheden and Ryde, 2015; Sgobba et al., 2012). Instead of simulating the system’s molecular dynamics over time, the Prime MM-GBSA module in the Schrodinger suite, performs a series of calculations on a single optimized pose from Glide’s docking output, using a Generalized Born solvation model (VSGB 2.0) (Li et al., 2011) to estimate the binding free energy. These calculations involve minimization to relax any strained interactions, energy calculation of the complex and its components using the MM-GBSA method, and finally, determining the binding affinity by calculating the difference in energies (E) of the protein-ligand’s complex and its components (Muddagoni et al., 2021):

$$\Delta G_{\text{bind}}=E{\_}_{\text{complex}\;\left(\text{minimized}\right)}\;–\;E{\_}_{\text{ligand}\;\left(\text{minimized}\right)}\;–\;E{\_}_{\text{receptor}\;\left(\text{minimized}\right)}$$

This approach offers significant time efficiency compared to standard MM-GBSA, which relies on lengthy MD simulations. However, it is important to note that this method is limited by the single, optimized docked pose, potentially missing the full range of conformations the ligand can adopt in the binding site and potentially neglecting entropic effects. Overall, Prime MM-GBSA without MD can be a useful tool for quick binding affinity estimation. However, for more accurate and complete understanding of the binding process, a full MD simulation is recommended. Therefore, in our study, the poses obtained with the highest docking score for each ligand across all 17 targets were re-scored using the Prime (Schrödinger, 2023a) MM-GBSA to calculate binding free energy (ΔG) in kcal/mol. Then, MD simulations were performed for the best scoring complex.

### Molecular dynamics (MD) simulations

Building upon the promising MM-GBSA and docking results, Complex **9**, which emerged as the top contender from a group of twenty Phosphinogold(I) Thiocarbohydrate Complexes, was subjected to detailed molecular dynamics (MD) simulations. The 100 ns simulations, performed using Desmond software (Schrödinger, 2018), aimed to explore the dynamic interactions between Complex **9** and its target proteins: Poly (ADP-ribose) polymerase (PARP-1, PDB ID 3L3M) and Protein Kinase B (AKT2, PDB ID 8Q61). To prepare the simulations, the protein-ligand complexes were placed in minimized, solvated orthorhombic boxes, ensuring a 10 Å buffer of TIP3P water molecules. The simulation environments were brought to physiological ionic strength by adding counter ions for neutralization and 0.15 M NaCl. The MD simulations were performed under NPT conditions (constant particle number, pressure, and temperature) at 300 K and 1.01325 bar, with the OPLS3e force field. Prior to the 100 ns production run, all systems were relaxed using Desmond’s default 160 picosecond protocol. Following the simulation, Maestro (Schrödinger, 2023b) was employed for comprehensive analysis of simulation data. Key parameters assessed included root mean square deviation (RMSD), root mean square fluctuation (RMSF), radius of gyration (Rg), the dynamic protein-ligand contact profiles, and finally, the calculation of post-simulation MM-GBSA binding free energy (ΔGbind in kcal/mol).

## Results

### Docking validation and evaluation of complex interactions

The reliability of the molecular docking protocol was established through redocking of native ligands into their respective protein structures, confirming accurate reproduction of experimentally observed binding poses (Figure 2; Table 2). Root mean square deviation (RMSD) was calculated to quantify the difference in position between co-crystallized and re-docked native ligands. An RMSD value equal to or less than 2.0 Å was used as a criterion to validate the docking protocol (Hevener et al., 2009; Jain, 2008). Following validation, molecular docking was performed using Glide XP, and subsequently, the more rigorous Prime MM-GBSA method was employed to refine binding affinity estimations. A detailed breakdown of observations for each complex can be found in the supplementary materials (Supplementary Tables S1–20; Supplementary Figurea S1–14).

### Molecular docking and prime MM-GBSA analysis

**FIGURE 3:**
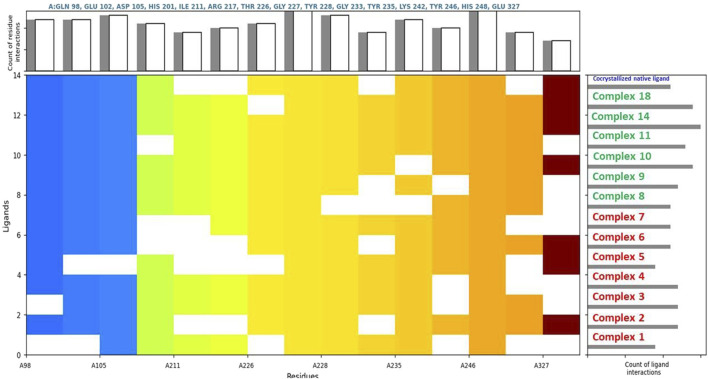
Comparative ligand-residue interaction profiles for thirteen Phosphinogold(I) Thiocarbohydrate complexes and the native ligand (indicated at the top right corner in blue) within the Poly (ADP-ribose) polymerase 1 (PARP1) binding site (PDB ID: 3L3M). The figure displays a matrix where the presence of an interaction between each ligand and a specific residue (A:GLN 98 – A:GLU 327) in PARP1 is indicated by a colored square. Colors are assigned arbitrarily and do not represent interaction strength or frequency. The total number of interactions per residue and per ligand are shown in the top and right panels, respectively, allowing for a comparative analysis of the interaction patterns. This figure clearly shows that binuclear complexes **8**–**20** have generally more interactions than mononuclear complexes **1** – **7**.

**FIGURE 4:**
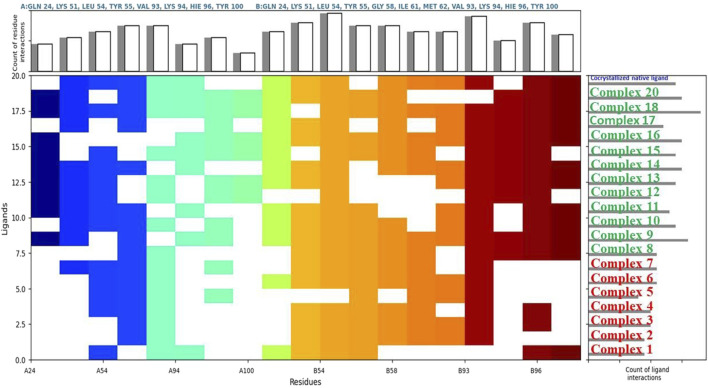
Comparative ligand-residue interaction profiles for nineteen Phosphinogold**(I)** Thiocarbohydrate complexes and the native ligand (indicated at the top right corner in blue) within the Human Double Minute 2 protein (HDM2) binding site (PDB ID: 5HMH). The figure displays a matrix where the presence of an interaction between each ligand and a specific residue (A:GLN 24 – A: TYR 100, and B:GLN 24 – B:TYR 100) in HDM2 is indicated by a colored square. Colors are assigned arbitrarily and do not represent interaction strength or frequency. The total number of interactions per residue and per ligand are shown in the top and right panels, respectively, allowing for a comparative analysis of the interaction patterns. This figure clearly shows that binuclear complexes **8**–**20** have generally more interactions than mononuclear complexes **1** – **7**.

The analysis identified several key protein targets, the most significant are Human Double Minute 2 protein (HDM2), DNA methyltransferase-1 (DNMT1), Human AKT2, and Poly (ADP-ribose) polymerase (PARP-1), which frequently exhibited strong interactions with multiple complexes. Number of complexes exhibited strong binding affinities, with several of them surpassing the binding strength of native ligands (Tables 3, 4). This suggests potential multi-targeting mechanisms and broad-spectrum activity. A general trend observed was that binuclear complex (those numbered 8 and above) consistently exhibited higher binding affinities than mononuclear complexes, as illustrated in Figures 3, 4. This suggests that the presence of two gold centers within the complex may be associated with enhanced binding. This relationship was confirmed by a statistically significant (α = 0.05) negative correlation between complex type (mononuclear or binuclear) and docking scores with two targets; DNMT-1 (Spearman’s correlation coefficient r = −0.837, p = 0.0061) and HDM2 (r = −0.717, p = 0.0242). The negative correlation indicates that binuclear complexes, which correspond to lower docking scores, bind more tightly to the proteins (Supplementary Tables S21, 22). The noted increase in binding affinity with binuclear complexes is entirely consistent with the results of Adokoh et al., who found that dinuclear gold(I) complexes (8-20) displayed significantly enhanced growth inhibition of cancer cells when compared to their mononuclear counterparts (1-7). Acetylation has been found to have a significant effect on binding affinity, with acetylated complexes (complexes **5**-**7** and **12**-**20**) consistently exhibiting higher docking scores (lower binding affinity) (as detailed in Supplementary Tables S1, 20) than their non-acetylated analogs (complexes **1-4** and **8**-**11**). This observation is supported by statistically significant positive correlations between acetylation and docking scores for DNMT-1 (Spearman’s r = 0.8452, p = 0.0016), HDM2 (r = 0.8563, p = 0.0001), PARP-1 (r = 0.8452, p = 0.0016), and AKT2 (r = 0.7681, p = 0.0081) (Supplementary Tables S22, 23). These positive correlations indicate that acetylation is associated with higher (less negative) docking scores, suggesting a weaker binding affinity. While this reduction in binding affinity can be explained by reduced hydrogen bond forming capability, Adokoh et al. observed that acetylation paradoxically enhanced the activity of gold(I) complexes (specifically complexes **5**-**7** in their study). This difference between the *in silico* finding and the experimental *in vitro* activity underscores that acetylation has a complex and multifaceted effect, where its impact on binding affinity does not necessarily correlate directly with its impact on overall cellular activity due to factors such as cell penetration, which Adokoh et al. attributed to increased lipophilicity as a result of reduced hydrogen bonding potential. The following illustrative examples underscore the significant interactions observed in this study. Table 4 provides a complete listing of the strongest interactions.

**TABLE 3:** Compounds with the highest binding free energy across the 17 cancer targets.

Complexes	Target PDB ID	MM-GBSA Kcal/mol	Target description
9	8Q61	−82.4	Human AKT2
5	5HMH	−76.0	Human Double Minute 2 protein (HDM2)
9	3L3M	−75.7	Poly (ADP-ribose) polymerase (PARP1)
13	3PP0	−71.1	Human epidermal growth factor receptor 2 (HER2)
1	4WXX	−69.9	DNA methyltransferase_1
15	8HOI	−64.8	B-cell lymphoma 2 (BCL2)
11	1JFF	−62.2	Alpha-Beta-tubulin dimer
17	3RCD	−59.5	Human epidermal growth factor receptor 2 (HER2)
3	4XV2	−59.0	B-Raf Kinase
10	1JDH	−58.0	β-catenin
7	1HCK	−53.5	Human cyclin-dependent kinase 2
12	3D0E	−51.1	Human Akt2
12	1M17	−49.8	Epidermal Growth Factor Receptor (EGFR)
3	2GU8	−43.9	Akt/protein kinase B
10	4JT5	−39.1	Mammalian target of rapamycin (mTOR)
7	4OBE	−21.2	GDP-bound Human KRas
5	1O6L	−6.30	Activated Akt/protein kinase B

**TABLE 4:** Free binding energies of the top 30 interactions compared to native ligands.

No.	Target PDB ID	Target description	Complexes	MM-GBSA kcal/mol	Cocrystallized ligand/inhibitor MM-GBSA
^∗∗^1	8Q61	Human AKT2	**9**	-82.435	-59.273
2	5HMH	HDM2	**5**	-76.030	-94.448
^∗∗^3	3L3M	PARP1	**9**	-75.683	-69.321
^∗∗^4	3L3M	PARP1	**8**	-73.429	-69.321
5	3PP0	HER2	**13**	-71.146	-91.763
^∗∗^6	4WXX	DNA methyltransferase_1	**1**	-69.886	-68.598
7	5HMH	HDM2	**3**	-67.408	-94.448
8	4WXX	DNA methyltransferase_1	**10**	-66.491	-68.598
9	5HMH	HDM2	**8**	-66.290	-94.448
10	5HMH	HDM2	**14**	-66.120	-94.448
11	4WXX	DNA methyltransferase_1	**9**	-65.857	-68.598
12	4WXX	DNA methyltransferase_1	**8**	-65.478	-68.598
13	5HMH	HDM2	**4**	-65.055	-94.448
14	8HOI	BCL2	**15**	-64.831	-104.576
^∗∗^15	8Q61	Human AKT2	**3**	-64.084	-59.273
16	1JFF	Alpha-Beta-tubulin dimer	**11**	-62.171	-77.743
17	5HMH	HDM2	**7**	-60.645	-94.448
18	3RCD	HER2	**17**	-59.549	-70.002
19	3L3M	PARP1	**5**	-59.508	-69.321
20	4XV2	B-Raf Kinase	**3**	-58.954	-69.397
21	3L3M	PARP1	**2**	-58.717	-69.321
22	5HMH	HDM2	**6**	-58.125	-94.448
^∗∗^23	1JDH	β-catenin	**10**	-58.035	-28.646
24	5HMH	HDM2	**20**	-56.791	-94.448
25	3L3M	PARP1	**7**	-56.790	-69.321
26	5HMH	HDM2	**2**	-56.759	-94.448
27	8HOI	BCL2	**9**	-56.711	-104.576
28	8HOI	BCL2	**6**	-56.297	-104.576
29	4XV2	B-Raf Kinase	**1**	-56.235	-69.397
30	4XV2	B-Raf Kinase	**8**	-55.938	-69.397

**Highlighted rows indicate complexes with binding free energy higher than the cocrystallized ligands.

**FIGURE 5:**
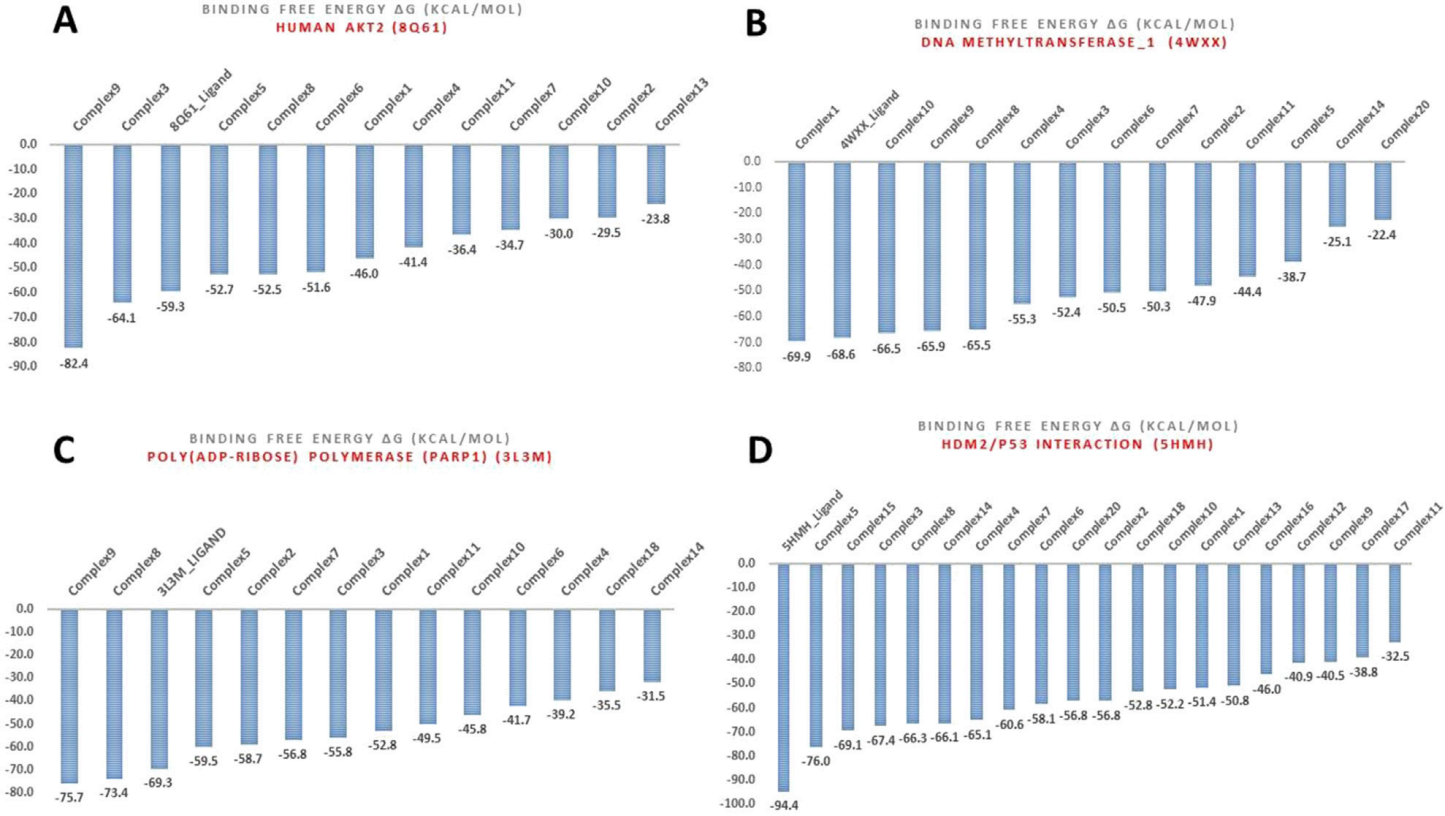
MM-GBSA binding free energies (Kcal/mol) for the various complexes with targets: **(A)** Human AKT2 (8Q61), **(B)** DNA methyltransferase-1 (DNMT1) (4WXX), **(C)** Poly (ADP-ribose) polymerase (PARP1) (3L3M), and **(D)** Human Double Minute 2 protein (HDM2) (5HMH).

Complex **9:** This complex stood out as a top performer against Human AKT2 isoform 8Q61, displaying an MM-GBSA binding score of −82.4 kcal/mol (Supplementary Table S9; Figure 5C), which exceeded even the native ligand’s score of −59.3 kcal/mol. This strong interaction aligns well with the strong anti-proliferative effects that have been seen with complexes **8**-**11** *in vitro*, and suggests that disruption of AKT2 may be a key factor in its success. Complex **9** also demonstrated strong affinity for PARP1 with MM-GBSA score of −75.7 kcal/mol and was therefore, considered for further MD study. Unfortunately, complex **9** was selected for *in vitro* studies at the time and however, it is imperative to further investigate complex **9**. Complex **5** showed the highest binding affinity towards the HDM2 with a binding free energy of −76.0 kcal/mol. Several other complexes also showed promising interactions with HDM2, this suggests that inhibiting 5HMH/p53 interaction plays a crucial role in the mechanism of action of these complexes. Complex **10,** demonstrated multi-target binding interactions, in particular HDM2, DNMT-1, and a particular affinity towards β-catenin (1JDH), were exceeding the MM-GBSA score of the reference inhibitor by a notable margin (Table 4). The tight binding of complex **10** with beta-catenin is crucial in the Wnt signaling pathway (Zhao et al., 2022) which supports its potent activity in colon cancer (HCT116 cell line) *in vitro* (IC_50_ = 0.90 µM). Similarly, complex **11**, also displayed a diverse array of binding targets, notably with DNMT-1, PARP-1, and the strongest binding affinity observed for the Alpha-Beta-tubulin dimer (1JFF) which could also account for strong *in vitro* anti-proliferative effects against colon and prostate cancer with IC_50S_ of 0.63 µM and 0.22 µM respectively (Adokoh et al., 2017). Complex **8** being the shorter chain of **9** and **11** demonstrated a significant interaction with both DNMT-1 and HDM2, surpassing the respective native ligand of PARP1 in line with *in vitro* data, which was the most potent growth inhibitor of prostate (PC3) cell line (IC_50_ = 0.003 μM) (Adokoh et al., 2017).

### MD simulations

**FIGURE 6:**
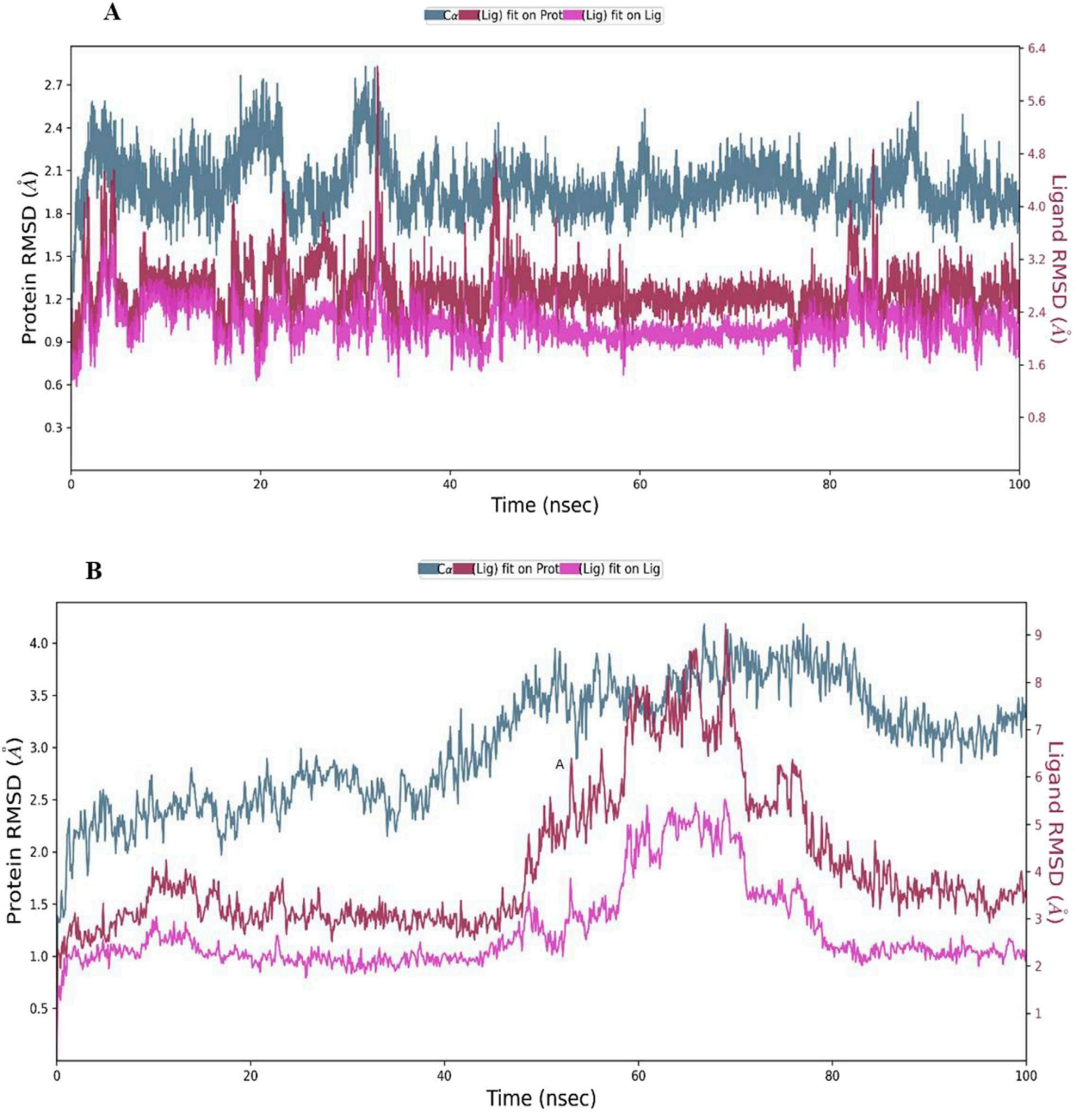
Dynamic Stability of complex **9** with **(A)** PARP-1 (3L3M) and **(B)** AKT2 (8Q61) during MD Simulation. This figure visualizes the root mean square deviation (RMSD) of the proteins and Complex **9**, over the 100 ns MD simulation. The plot shows three RMSD traces: dark blue for the protein’s C-alpha atoms, red for Complex **9** fitted to the protein (highlighting the ligand’s movement within the binding pocket), and pink for Complex **9** fitted to itself (demonstrating its conformational flexibility).

Complex **9**’s binding stability with PARP-1 and AKT2 was investigated via 100 ns MD simulations. As shown in Figure 6A and Table 5, the PARP-1-Complex **9** system achieved equilibrium after approximately 10 ns, exhibiting a stable protein Cα RMSD averaging 2.0 Å (range: 1.8–2.4 Å). This suggests minimal overall protein structural fluctuation. While the ligand RMSD relative to the protein (ligand fitted on protein) showed some fluctuations, reaching up to ∼6 Å in few instances, it was generally stabilized between 2.0 and 3.0 Å, averaging 2.72 Å. This suggests sustained binding despite some ligand mobility within the pocket, likely due to conformational adjustments. The ligand’s internal structure remained rigid, as evidenced by the low average RMSD of 2.21 Å (range: 1.6–2.5 Å) when fitted on itself. In contrast, the AKT2-Complex **9** simulation (Figure 6B) revealed a less stable protein Cα RMSD profile, although equilibrium was reached around 20 ns with an average of 3.03 Å (Table 5). Ligand mobility was more pronounced in this system, with an average ligand RMSD (relative to the protein) of 4.13 Å (Table 5). A period of increased fluctuation between 46 and 76 ns suggests substantial ligand conformational changes during this timeframe, potentially impacting the binding site and correlating with the observed jump in protein RMSD (Figure 6B). Despite this increased mobility relative to the protein, the ligand’s internal structure remained relatively rigid, averaging 2.74 Å RMSD when fitted on itself. This indicates the ligand’s conformational changes involve shifts in position or orientation within the binding pocket rather than significant internal rearrangements.

**TABLE 5:** ^**^Average structural and dynamic analysis of complex **9** during MD Simulations.

Target	RMSD C-alpha	RMSD Ligand fitted on Protein	RMSD Ligand fitted on Ligand	rGyr	MolSA	SASA	PSA
PARP-1	2.00	2.72	2.21	7.82	836.06	428.67	422.81
AKT2	3.03	4.13	2.74	7.02	800.27	457.71	410.57

RMSD: Root mean square deviation (Å) rGyr: Radius of gyration (Å).

MolSA: Molecular surface area (Å^2^).

SASA: Solvent accessible surface area (Å^2^).

PSA: Polar surface area (Å^2^).

**Values are average calculated from 1,000 frames during 100ns simulations.

**FIGURE 7:**
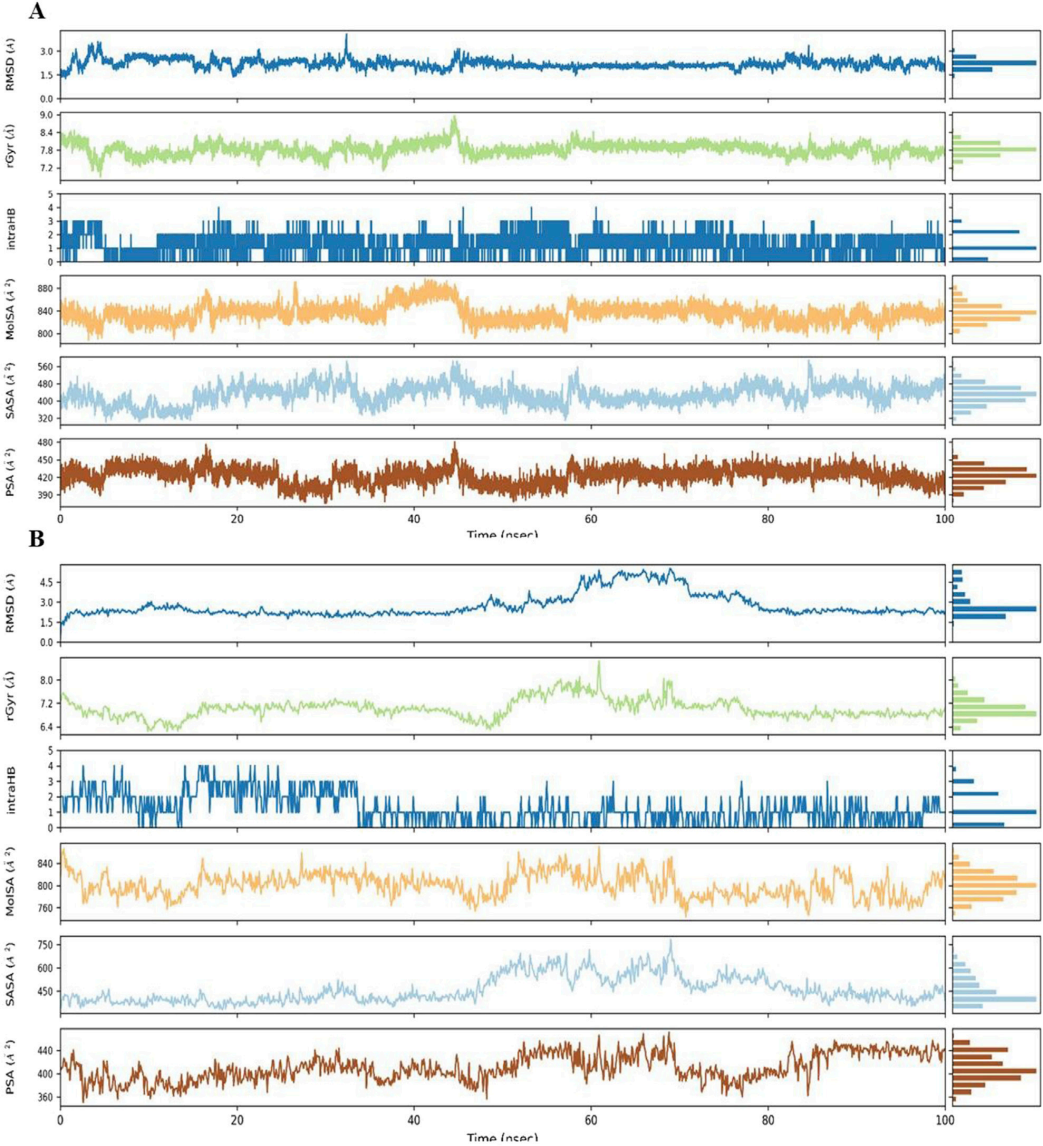
The figure displays the dynamic behavior of Complex **9** within the binding site of **(A)** PARP-1 and **(B)** AKT2, tracking changes in Root Mean Square Deviation (RMSD), radius of gyration (rGyr), intramolecular hydrogen bonds (intraHB), Molecular Surface Area (MolSA), Solvent Accessible Surface Area (SASA), and Polar Surface Area (PSA). Each parameter (with units of Å or Å^2^) is shown as a trace across the simulation time with a histogram of the parameter’s distribution.

Figure 7 summarizes the properties of complex **9** bound to (A) PARP-1 and (B) AKT2 during MD simulations, including radius of gyration (rGyr), molecular surface area (MolSA), solvent accessible surface area (SASA), and polar surface area (PSA). Complex **9** displayed a more compact average structure and reduced solvent exposure with AKT2 (average rGyr ≈7.0 Å, range 6.2–8.6 Å; average SASA ≈458 Å^2^, range 330–783 Å^2^) compared to PARP-1 (average rGyr ≈7.8 Å, range 6.8–8.9 Å; average SASA ≈428 Å^2^, range 300–590 Å^2^), although the PARP-1 system showed greater rGyr fluctuation. Intramolecular hydrogen bonding was similarly low and variable in both simulations (average <2, range 0–4 H-bonds), indicating it is unlikely to be a key stabilizing factor. Polar surface area values were comparable (≈411 Å^2^ for AKT2 and ≈423 Å^2^ for PARP-1). These results indicate Complex **9** binds more dynamically to AKT2 than to PARP-1. Table 5 also summarizes the average values for these properties.

**FIGURE 8:**
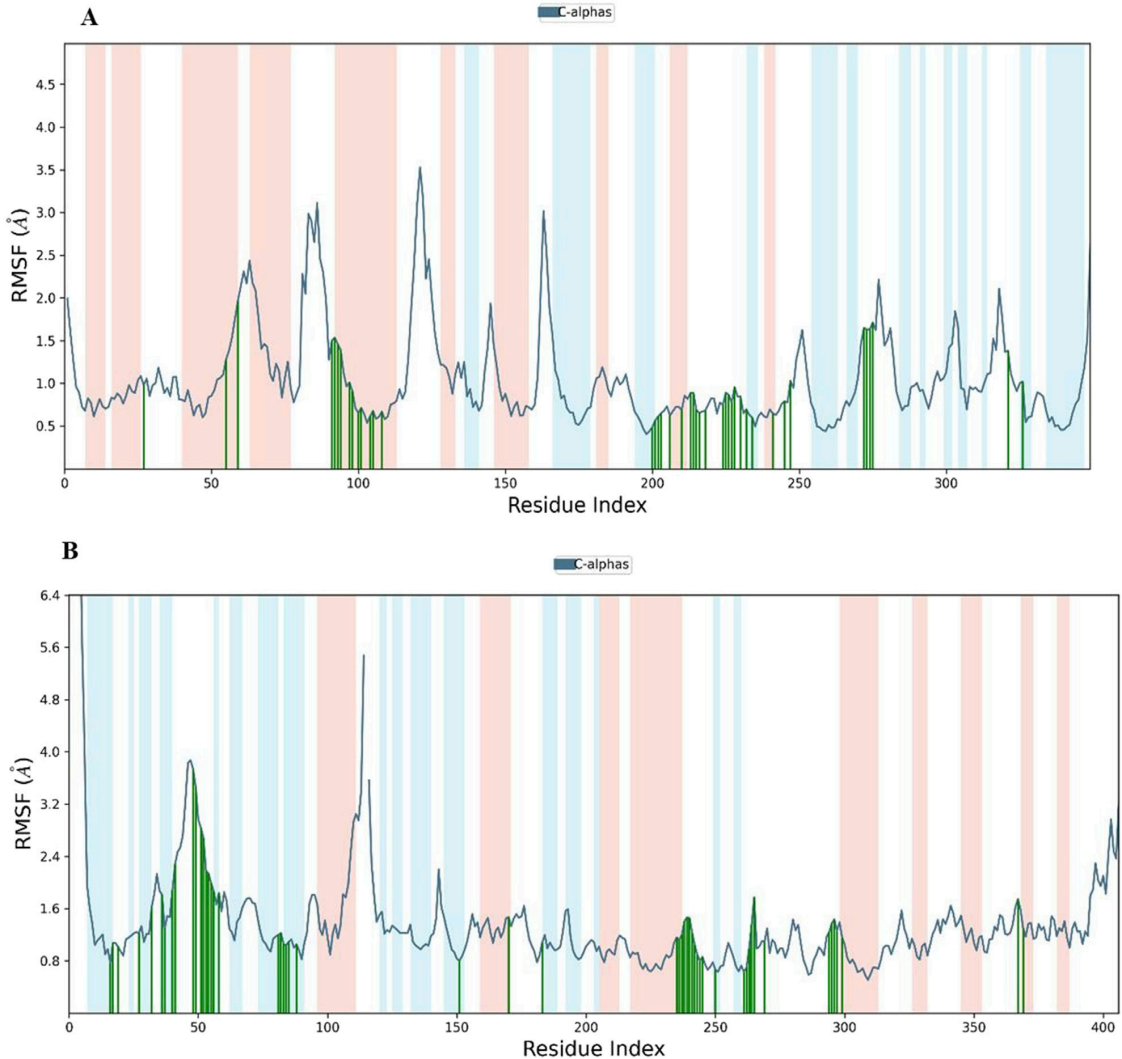
Root Mean Square Fluctuation (RMSF) of **(A)** PARP-1 and **(B)** AKT2 in complex with complex **9**. The blue line shows the RMSF of the protein’s C-alpha atoms. Green vertical lines indicate residues interacting with Complex **9**, and the shaded background highlights the protein’s secondary structure elements (red for alpha helices and light blue for beta strands).

**FIGURE 9:**
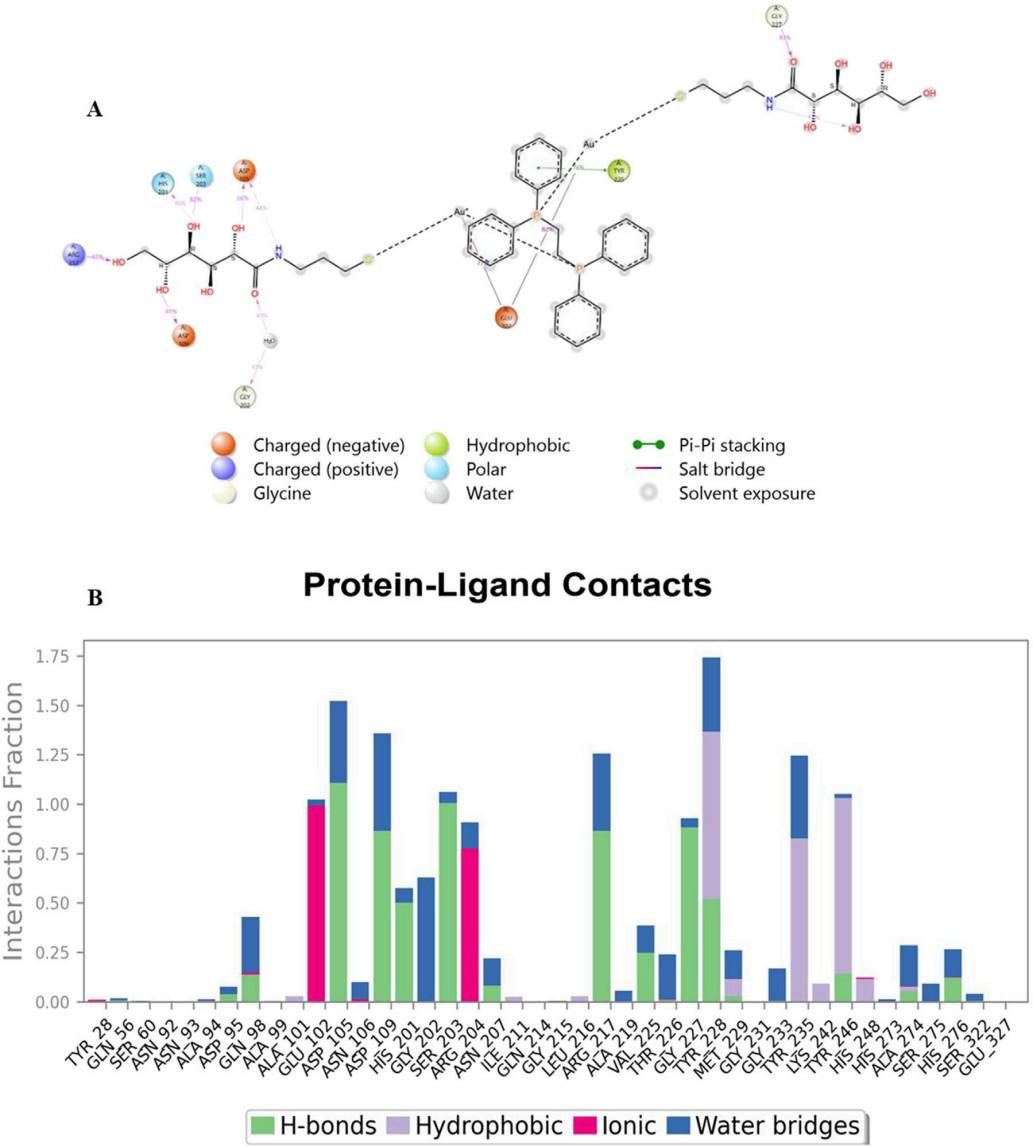
Interaction profile of complex **9** with PARP-1 binding site residues. Panel **(A)** displays a 2D interaction diagram, highlighting the specific contacts (ionic interactions, hydrogen bonds, hydrophobic interactions, and water bridges) between Complex **9** and key PARP-1 residues. Panel **(B)** shows an interaction fraction plot, quantifying the frequency with which each residue interacts with Complex **9** across the molecular dynamics simulation.

**FIGURE 10:**
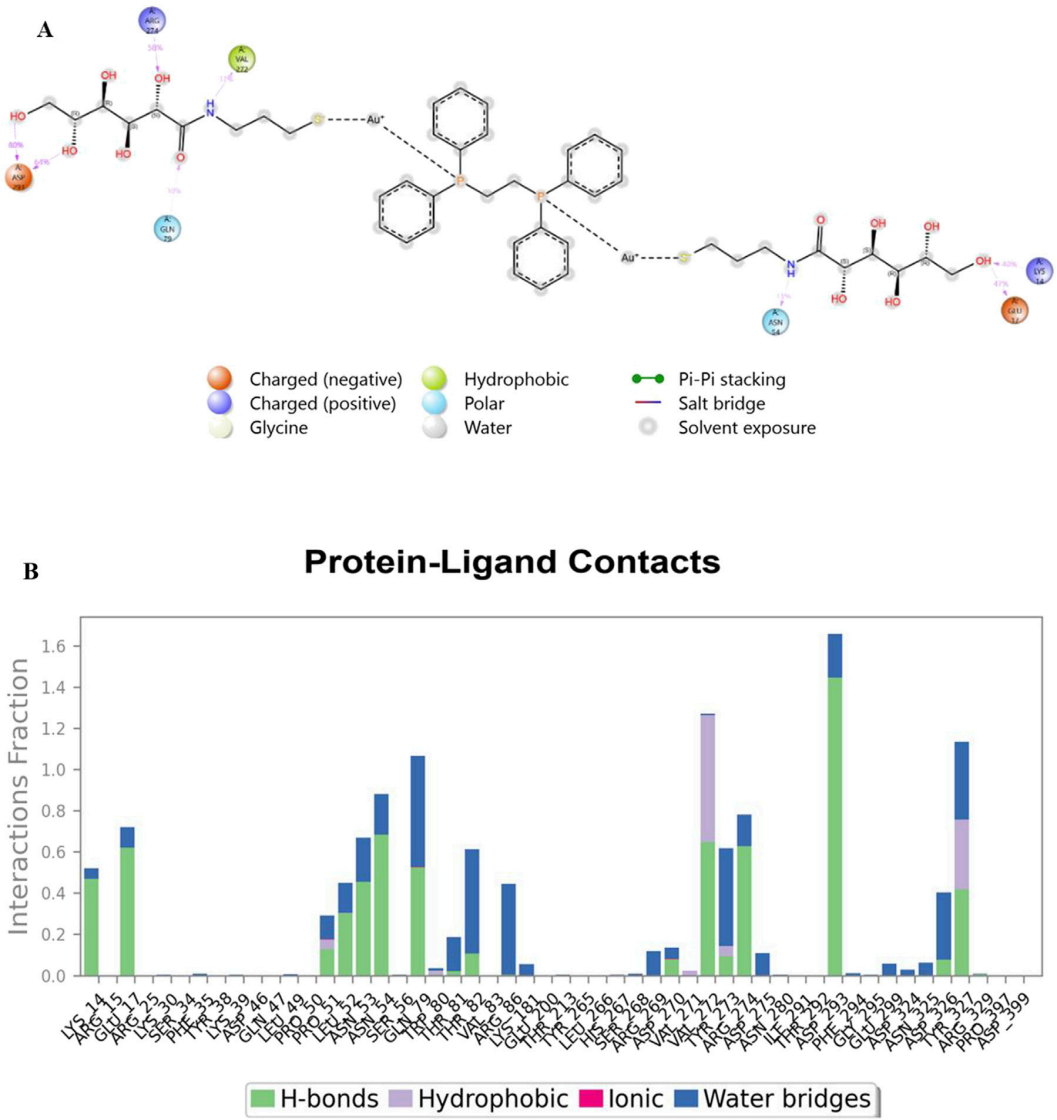
Interaction profile of complex **9** with AKT2 binding site residues. Panel **(A)** displays a 2D interaction diagram, highlighting the specific contacts (ionic interactions, hydrogen bonds, hydrophobic interactions, and water bridges) between Complex **9** and key AKT2 residues. Panel **(B)** shows an interaction fraction plot, quantifying the frequency with which each residue interacts with Complex **9** across the molecular dynamics simulation.

Analysis of Cα RMSF for PARP-1 bound to complex **9** (Figure 8A) suggests that ligand binding modulates protein flexibility. While the N- and C-termini appear generally rigid, ligand contacts at various locations, including residues 27-28, 55-60, 92-109, 200-275, and 322, may contribute to localized changes in flexibility. For example, increased RMSF values around residues 57-64 could indicate ligand-induced conformational changes. It is important to acknowledge that these observations are based on a single MD simulation and require further validation, including comparison to apo PARP-1 dynamics and experimental studies, to definitively establish the effects of complex **9** binding. On the other hand, complex **9** binding differentially affects the flexibility of AKT2. PARP-1’s N-terminus becomes rigid upon binding, whereas AKT2’s N-terminus shows a substantial increase in flexibility (i.e., higher RMSF) (Figure 8B). Overall, complex **9** has a more dramatic effect on AKT2’s flexibility, particularly at the N- and C-termini. In contrast, complex 9’s impact on PARP-1 is more localized. AKT2 also displays a greater number of contact points with complex **9**. This broader interaction interface may suggest a more extensive allosteric effect of complex **9** on AKT2 compared to PARP-1. Further studies are needed to validate how these distinct dynamic responses relate to the functional regulation of each protein. Figures 9, 10 illustrate the interaction profiles of complex **9** with PARP-1 and AKT2 binding site residues, respectively. The prominent mode of interaction in both cases is through H-bonding and to a lesser extent, water bridges, which is a special form of H-bonds mediated by surrounding water molecules. This explain the higher affinities of non-acetylated series of complexes such as complex **9**, compared to those of the per-acetylated complexes such as **12**-**20** as we have discussed earlier.

**FIGURE 11:**
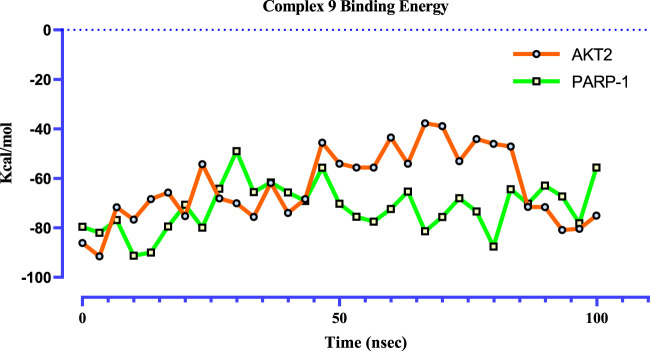
Time-dependent binding free energy profiles of complex **9** with PARP1 (green) and AKT2 (orange), computed via the MM-GBSA method from 100 ns molecular dynamics trajectories (n = 31). The calculated average binding free energies were −71.82 ± 3.65 kcal/mol for PARP1 and -63.31 ± 5.33 kcal/mol for AKT2. These values suggest a significantly more thermodynamically favorable binding between complex **9** and PARP1, implying a stronger and more stable interaction relative to AKT2.

Prime MM-GBSA calculations, using 31 frames extracted at ∼3.3 ns intervals from 100 ns MD trajectories (Figure 11), provide a more comprehensive understanding of the binding profile of complex **9** with PARP-1 and AKT2 compared to previous calculations based on the single docked poses in the molecular docking study. These post-MD calculations, incorporating protein and ligand flexibility by sampling representative snapshots across the simulation, offer a more realistic representation of the binding dynamics. Complex **9** displays a more favorable and stable binding profile with PARP-1, exhibiting an average binding free energy of −71.82 kcal/mol and fluctuations mostly within a −50 to −91 kcal/mol range. In contrast, complex **9** binding to AKT2 is characterized by a lower average binding free energy (−63.31 kcal/mol) and wider fluctuations (−38 to −91 kcal/mol), suggesting a weaker or more dynamic interaction.

## Discussion

The integration of *in silico* and *in vitro* data underscores the significance of computational approaches in identifying promising lead compounds and elucidating their mechanisms of action. In this study, *in silico* analyses revealed substantial interactions between phosphinogold(I) thiocarbohydrate complexes and key proteins implicated in cancer progression. These interactions suggest mechanisms targeting cell survival, proliferation, DNA repair, and apoptosis. Notably, complexes **5, 8, 9, 10**, and **11** exhibited strong binding to HDM2, DNMT1, AKT2, and PARP-1, indicating a multi-target strategy that could enhance therapeutic efficacy by simultaneously modulating several signaling pathways.

Among these interactions, the binding to HDM2 is particularly significant, given its role in regulating the tumor suppressor protein p53 (Wang et al., 2023). Overexpression of HDM2 in cancer inhibits p53 function, leading to uncontrolled cell growth (Nag et al., 2014; Wang et al., 2023; Yang et al., 2002). The *in silico* findings suggest that these complexes, particularly complex **5**, may disrupt the HDM2/p53 interaction, thereby restoring p53 activity and promoting tumor suppression. Similarly, complexes **1, 8, 9**, and **10** demonstrated strong interactions with DNMT1 (Figure 5B), an enzyme responsible for maintaining DNA methylation patterns (Chen et al., 2010; Massie et al., 2017; Mohd Kamal et al., 2024). Aberrant methylation can silence tumor suppressor genes, and inhibition of DNMT1 by these complexes may restore their expression, thereby suppressing cancer cell proliferation (Mohd Kamal et al., 2024).

AKT2, a key player in the PI3K/AKT/mTOR signaling pathway, is frequently dysregulated in cancers (Attoub et al., 2022), promoting tumor growth and therapeutic resistance (Chau and Ashcroft, 2004; Riggio et al., 2017; Rychahou et al., 2008; Su et al., 2021) The strong binding of complex **9** to AKT2 suggests its potential to disrupt this pathway, thereby inhibiting tumor proliferation. Furthermore, complex **9** exhibited a significant affinity for PARP-1, an enzyme involved in DNA damage repair (Bondar and Karpichev, 2024; Deshmukh and Qiu, 2015; Puentes-Pardo et al., 2023). Given the established efficacy of PARP inhibitors in targeting cancers with defective DNA repair mechanisms, complex **9** may potentiate anti-cancer effects by disrupting PARP-1 activity (Bondar and Karpichev, 2024).

To further characterize complex **9**, molecular dynamics simulations were performed with PARP-1 and AKT2, revealing distinct binding dynamics. Complex **9** demonstrated a stable interaction with PARP-1, as indicated by lower RMSD values and a narrower range of MM-GBSA binding free energies, suggesting a more favorable binding profile. Conversely, its interaction with AKT2 was more dynamic, characterized by higher RMSD and RMSF values, particularly at the termini, and a broader range of MM-GBSA energies. These findings suggest that complex **9** preferentially stabilizes PARP-1 binding, warranting further experimental validation to assess its therapeutic potential.

The *in silico* findings were correlated with *in vitro* observations from Adokoh et al., where complexes with high binding affinities to key targets exhibited potent activity in cell-based assays (Table 6). A critical consideration in ligand design is the balance between lipophilicity, modulated by acetylation, and direct binding affinity. While acetylation enhances cellular uptake by increasing lipophilicity, our findings suggest that it may simultaneously reduce direct binding affinity by limiting hydrogen bonding interactions. Docking and MM-GBSA analyses revealed that non-acetylated complexes (**8, 9, 10**, and **11**) exhibited the strongest interactions, emphasizing the importance of free hydroxyl groups in target binding. However, the acetylated complex **5 and 13**, for example, demonstrated strong binding affinity, suggesting a nuanced balance between hydrogen bonding and lipophilicity. Complex **13**, for example, typically showed high binding affinity (−71.1 kcal/mol) to HER2, collaborating the high *in vitro* activity against prostate, colon and breast cancer cell lines (IC_50_ = 0.03, 0.25, and 0.07 µM respectively) reported by our group. Strangely, no statistically significant correlation was found for complex 13 in this work and the *in vitro* work by Adokoh et al. (2017). These results indicate that a uniform acetylation strategy may not be optimal for all target proteins.

**TABLE 6:** Summarizing and linking some *in silico* findings to the *in vitro* data from the studies of Adokoh et al.

Complexes	*In Silico* Key Findings in this work	*In Vitro* Observations (Adokoh et al., 2017)	Correlation/Insights
8	Strong binding to PARP1 (−73.43 kcal/mol) DNMT-1 (-65.48 kcal/mol) and HDM2 (−66.29 kcal/mol)	Exhibited the lowest IC_50_ (0.003 µM against PC3 cells)	The potent *in vitro* activity aligns with high affinity for DNMT-1 and HDM2, targets critical for PC3 survival. The affinity to PARP1 will reverse especially, BRCA1/BRCA2-mutated triple-negative breast cancer (TNBC) (Dilmac and Ozpolat, 2023)
5	Promising interaction with HDM2 (−76.03 kcal/mol)	Enhanced activity with lower selectivity. MCF7 (1.94 µM), and PC3 (2.20 µM)	HDM2 is a negative regulator of p53, a tumor suppressor protein that prevents uncontrolled cell division (Wang et al., 2023). Targeting HER2-positive and triple-negative subtypes will halt HDM2 overexpression correlates with poor prognosis leading to breast cancer treatment
10	Strong binding to β-catenin and DNMT-1 (−66.491 kcal/mol)	Potent activity against, PC3 (0.08 µM) and HCT116 cells (0.90 µM)	High affinity for DNMT-1, targets critical for PC3 survival (Tzelepi et al., 2020) and Tight β-catenin binding aligns with its role in the Wnt signaling pathway (Zhao et al., 2022), crucial for colon cancer
13	Highly affinity for HER2 (−71.1 kcal/mol)	Potent activity against, MCF7 (0.70 µM), PC3 (0.03 µM) and HCT116 cells (0.25 µM)	The potential *in vitro* activities aligns with high affinity for HER2 critical for breast and other cancers (Cheng, 2024; Rubin et al., 2024). Thus, in HER2-positive and triple-negative subtypes, HDM2 overexpression correlates with poor prognosis leading to breast cancer. But no correlation was found
14	Moderate affinity for HDM2 (−66.12 kcal/mol) and broad but weaker binding across targets	Higher activity but reduced tumor specificity compared to earlier analogs. MCF7 (0.14 µM), PC3 (0.84 µM) and HCT116 cells (0.14 µM)	The potential *in vitro* activities aligns with high affinity for HDM2 critical for breast and prostate cancers. HDM2 inhibitors (e.g., Nutlin-3, RG7112, Idasanutlin) are being explored as cancer treatments (Alaseem, 2023), particularly in tumors with wild-type p53, as they can restore p53 function and induce apoptosis in cancer cells

Future research should focus on systematically optimizing acetylation patterns to enhance both binding affinity and cellular permeability. Strategies may include: (1) exploring partial acetylation to modulate lipophilicity while preserving hydrogen bonding, (2) incorporating alternative modifications to improve permeability without compromising target binding, (3) screening libraries of analogs with varying acetylation degrees to assess their impact on binding affinity and cellular activity, and (4) developing computational models to predict ligand permeability and interaction strength. Moreover, *in vitro* and *in vivo* validation, particularly of complex **9**, is crucial to confirm its therapeutic potential. These investigations could identify an optimal balance between acetylation and binding efficacy, thereby guiding the rational design of future anticancer agents.

## Conclusion

This *in silico* investigation provides compelling evidence for the anticancer potential of phosphinogold(I) thiocarbohydrate complexes, particularly complex **9**. The study highlights a multi-target mechanism, with strong interactions observed against key protein targets in cancer pathways, including HDM2, DNMT1, AKT2, and PARP-1. The correlation between *in silico* binding affinities and previously reported *in vitro* activity strengthens the validity of our computational approach. Molecular dynamics simulations further differentiated the binding dynamics of complex **9** with PARP-1 and AKT2, revealing a more stable interaction with PARP-1. This emphasizes PARP-1 as a particularly promising target for complex **9** and warrants further investigation and experimental validation. Furthermore, our findings underscore the importance of balancing lipophilicity, influenced by acetylation, with target binding affinity in future ligand design efforts. This research lays the groundwork for the development of more effective and selective gold-based anticancer therapies.

## Data Availability

The datasets presented in this study can be found in online repositories. The names of the repository/repositories and accession number(s) can be found in the article/Supplementary Material.
