# Supplementary material for: In silico molecular studies of Phosphinogold(I) thiocarbohydrate complexes: insights into multi-target anticancer mechanisms
Source: Front Chem. 2025 Jun 12;13:1533026. doi: 10.3389/fchem.2025.1533026 (PMC12198200; doi:10.3389/fchem.2025.1533026)
Supplement: Supplementary file 1 [file DataSheet1.docx]

***In Silico* Molecular Studies of** **Phosphinogold(I) Thiocarbohydrate Complexes**

Alkhair Adam Khalil Mohamed^1^, Isaac Asiamah^2^, James Darkwa^3^ and Christian K. Adokoh^4^^[[1]](#footnote-1)^

^1^Department of Biomedical Sciences, School of Allied Health Sciences, College of Health and Allied Sciences, University of Cape Coast, Cape Coast, Ghana

^2^Department of Chemistry, School of Physical Sciences, College of Agriculture and Natural Sciences, University of Cape Coast, Cape Coast, Ghana

^3^Department of Chemical Sciences, University of Johannesburg, Auckland Park, 2006, South Africa

^4^Department of Forensic Science, School of Biological Sciences, College of Agriculture and Natural Sciences University of Cape Coast, Cape Coast, Ghana

Table S1. Molecular docking scores (kcal/mol) and binding free energy of **complex1** to the 17 different cancer targets.

| Compound | Docking score kcal/mol | MM-GBSA  Kcal/mol | Target PDB ID | Target description |
| --- | --- | --- | --- | --- |
| Complex1 | -11.641 | -69.9 | 4WXX | DNA methyltransferase_1 |
| Complex1 | -11.170 | -51.4 | 5HMH | HDM2/p53 interaction |
| Complex1 | -9.655 | 9.2 | 1O6L | Activated Akt/protein kinase B |
| Complex1 | -9.348 | -52.8 | 3L3M | Poly (ADP-ribose) polymerase (PARP-1) |
| Complex1 | -8.858 | -13.0 | 2GU8 | Akt/protein kinase B |
| Complex1 | -8.644 | -46.0 | 8Q61 | Human Akt2 |
| Complex1 | -8.022 | -56.2 | 4XV2 | B-Raf Kinase |
| Complex1 | -7.644 | 39.8 | 1HCK | Human cyclin-dependent kinase 2 |
| Complex1 | -7.564 | -47.0 | 1JDH | β-catenin and HTCF-4 |
| Complex1 | -7.418 | -33.6 | 3RCD | Kinase domain of human epidermal growth factor receptor 2 (HER2) |
| Complex1 | -6.659 | 65.3 | 4OBE | GDP-bound Human KRas |
| Complex1 | -6.609 | -55.3 | 8HOI | B-cell lymphoma 2 (BCL2) |
| Complex1 | -6.210 | -37.2 | 4JT5 | Mammalian target of rapamycin (mTOR) |
| Complex1 | -6.126 | -44.3 | 1JFF | Alpha-Beta-tubulin dimer |
| Complex1 | -6.080 | 2.8 | 3D0E | Human AKT2 |
| Complex1 | -5.958 | -43.0 | 1M17 | Epidermal Growth Factor Receptor (EGFR) tyrosine kinase domain |
| Complex1 | -5.539 | -33.8 | 3PP0 | Kinase domain of human epidermal growth factor receptor 2 (HER2) |

Table S2. Molecular docking scores (kcal/mol) and binding free energy of **complex 2** to the 17 different cancer targets.

| Compound | Docking score kcal/mol | MM-GBSA  Kcal/mol | Target PDB ID | Target description |
| --- | --- | --- | --- | --- |
| Complex2 | -12.259 | -58.7 | 3L3M | Poly (ADP-ribose) polymerase (PARP-1) |
| Complex2 | -10.394 | -47.9 | 4WXX | DNA methyltransferase_1 |
| Complex2 | -9.890 | -56.8 | 5HMH | HDM2/p53 interaction |
| Complex2 | -9.165 | 38.2 | 1O6L | Activated Akt/protein kinase B |
| Complex2 | -8.613 | -29.5 | 8Q61 | Human Akt2 |
| Complex2 | -7.332 | 29.2 | 1HCK | Human cyclin-dependent kinase 2 |
| Complex2 | -6.895 | 48.8 | 4OBE | GDP-bound Human KRas |
| Complex2 | -6.844 | -34.9 | 4XV2 | B-Raf Kinase |
| Complex2 | -6.576 | -47.6 | 1JFF | Alpha-Beta-tubulin dimer |
| Complex2 | -6.122 | -55.2 | 3RCD | Kinase domain of human epidermal growth factor receptor 2 (HER2) |
| Complex2 | -6.114 | -41.7 | 8HOI | B-cell lymphoma 2 (BCL2) |
| Complex2 | -6.052 | -29.4 | 2GU8 | Akt/protein kinase B |
| Complex2 | -5.663 | -39.4 | 3PP0 | Kinase domain of human epidermal growth factor receptor 2 (HER2) |
| Complex2 | -5.536 | -22.2 | 3D0E | Human AKT2 |
| Complex2 | -5.407 | -38.9 | 4JT5 | Mammalian target of rapamycin (mTOR) |
| Complex2 | -4.842 | -37.9 | 1M17 | Epidermal Growth Factor Receptor (EGFR) tyrosine kinase domain |
| Complex2 | -3.004 | -35.1 | 1JDH | β-catenin and HTCF-4 |

Table S3: Molecular docking scores (kcal/mol) and binding free energy of **complex 3** to the 17 different cancer targets.

| Compound | Docking score kcal/mol | MM-GBSA  Kcal/mol | Target PDB ID | Target description |
| --- | --- | --- | --- | --- |
| Complex3 | -11.820 | -67.4 | 5HMH | HDM2/p53 interaction |
| Complex3 | -11.177 | -52.4 | 4WXX | DNA methyltransferase_1 |
| Complex3 | -9.631 | 25.0 | 1O6L | Activated Akt/protein kinase B |
| Complex3 | -9.225 | -43.9 | 2GU8 | Akt/protein kinase B |
| Complex3 | -8.477 | -64.1 | 8Q61 | Human Akt2 |
| Complex3 | -8.384 | -59.0 | 4XV2 | B-Raf Kinase |
| Complex3 | -7.696 | -34.0 | 1JFF | Alpha-Beta-tubulin dimer |
| Complex3 | -7.450 | -55.8 | 3L3M | Poly (ADP-ribose) polymerase (PARP-1) |
| Complex3 | -7.413 | -46.2 | 3RCD | Kinase domain of human epidermal growth factor receptor 2 (HER2) |
| Complex3 | -7.316 | -36.0 | 1JDH | β-catenin and HTCF-4 |
| Complex3 | -7.218 | -39.1 | 8HOI | B-cell lymphoma 2 (BCL2) |
| Complex3 | -6.515 | -40.1 | 3PP0 | Kinase domain of human epidermal growth factor receptor 2 (HER2) |
| Complex3 | -6.249 | -33.0 | 1M17 | Epidermal Growth Factor Receptor (EGFR) tyrosine kinase domain |
| Complex3 | -6.208 | 55.1 | 1HCK | Human cyclin-dependent kinase 2 |
| Complex3 | -5.879 | -38.6 | 4JT5 | Mammalian target of rapamycin (mTOR) |
| Complex3 | -5.751 | -20.3 | 3D0E | Human AKT2 |

Table S4. Molecular docking scores (kcal/mol) and binding free energy of **complex 4** to targets.

| Compound | Docking score kcal/mol | MM-GBSA  Kcal/mol | Target PDB ID | Target description |
| --- | --- | --- | --- | --- |
| Complex4 | -11.382 | -55.3 | 4WXX | DNA methyltransferase_1 |
| Complex4 | -9.891 | -65.1 | 5HMH | HDM2/p53 interaction |
| Complex4 | -9.227 | -48.8 | 4XV2 | B-Raf Kinase |
| Complex4 | -8.587 | -39.2 | 3L3M | Poly (ADP-ribose) polymerase (PARP-1) |
| Complex4 | -7.884 | -10.6 | 2GU8 | Akt/protein kinase B |
| Complex4 | -7.750 | -37.8 | 1JFF | Alpha-Beta-tubulin dimer |
| Complex4 | -7.135 | 29.4 | 1HCK | Human cyclin-dependent kinase 2 |
| Complex4 | -6.818 | -41.4 | 8Q61 | Human Akt2 |
| Complex4 | -6.668 | -47.9 | 8HOI | B-cell lymphoma 2 (BCL2) |
| Complex4 | -6.527 | -33.6 | 1JDH | β-catenin and HTCF-4 |
| Complex4 | -6.367 | -35.7 | 3RCD | Kinase domain of human epidermal growth factor receptor 2 (HER2) |
| Complex4 | -6.310 | 53.2 | 4OBE | GDP-bound Human KRas |
| Complex4 | -6.212 | -16.6 | 3D0E | Human AKT2 |
| Complex4 | -6.121 | 18.6 | 1O6L | Activated Akt/protein kinase B |
| Complex4 | -6.075 | -34.0 | 1M17 | Epidermal Growth Factor Receptor (EGFR) tyrosine kinase domain |
| Complex4 | -5.486 | -25.9 | 4JT5 | Mammalian target of rapamycin (mTOR) |
| Complex4 | -3.565 | -31.0 | 3PP0 | Kinase domain of human epidermal growth factor receptor 2 (HER2) |

Table S5. Molecular docking scores (kcal/mol) and binding free energy of **complex 5** to targets.

| Compound | Docking score kcal/mol | MM-GBSA  Kcal/mol | Target PDB ID | Target description |
| --- | --- | --- | --- | --- |
| Complex5 | -7.229 | -76.0 | 5HMH | HDM2/p53 interaction |
| Complex5 | -5.095 | -59.5 | 3L3M | Poly (ADP-ribose) polymerase (PARP-1) |
| Complex5 | -4.775 | -52.1 | 8HOI | B-cell lymphoma 2 (BCL2) |
| Complex5 | -4.691 | -34.0 | 4JT5 | Mammalian target of rapamycin (mTOR) |
| Complex5 | -3.677 | -25.3 | 1JFF | Alpha-Beta-tubulin dimer |
| Complex5 | -3.544 | -31.7 | 2GU8 | Akt/protein kinase B |
| Complex5 | -2.845 | -2.4 | 4OBE | GDP-bound Human KRas |
| Complex5 | -2.653 | 44.0 | 1HCK | Human cyclin-dependent kinase 2 |
| Complex5 | -1.984 | -43.1 | 1M17 | Epidermal Growth Factor Receptor (EGFR) tyrosine kinase domain |
| Complex5 | 0.311 | -52.7 | 8Q61 | Human Akt2 |
| Complex5 | 0.346 | -38.7 | 4WXX | DNA methyltransferase_1 |
| Complex5 | 2.375 | -25.2 | 3RCD | Kinase domain of human epidermal growth factor receptor 2 (HER2) |
| Complex5 | 2.524 | -23.3 | 3D0E | Human AKT2 |
| Complex5 | 2.712 | -6.3 | 1O6L | Activated Akt/protein kinase B |
| Complex5 | 7.399 | -38.8 | 1JDH | β-catenin and HTCF-4 |
| Complex5 | 7.586 | -31.6 | 4XV2 | B-Raf Kinase |
| Complex5 | 8.049 | -26.5 | 3PP0 | Kinase domain of human epidermal growth factor receptor 2 (HER2) |

`

Table S6. Molecular docking scores (kcal/mol) and binding free energy of **complex 6** to targets.

| Compound | Docking score kcal/mol | MM-GBSA  Kcal/mol | Target PDB ID | Target description |
| --- | --- | --- | --- | --- |
| Complex6 | -7.944 | -58.1 | 5HMH | HDM2/p53 interaction |
| Complex6 | -4.713 | -56.3 | 8HOI | B-cell lymphoma 2 (BCL2) |
| Complex6 | -4.703 | -50.5 | 4WXX | DNA methyltransferase_1 |
| Complex6 | -3.812 | 87.6 | 1O6L | Activated Akt/protein kinase B |
| Complex6 | -1.898 | -38.1 | 1JFF | Alpha-Beta-tubulin dimer |
| Complex6 | -1.293 | -27.0 | 3D0E | Human AKT2 |
| Complex6 | -0.922 | -43.3 | 3PP0 | Kinase domain of human epidermal growth factor receptor 2 (HER2) |
| Complex6 | 0.326 | -41.7 | 3L3M | Poly (ADP-ribose) polymerase (PARP-1) |
| Complex6 | 0.484 | -36.7 | 2GU8 | Akt/protein kinase B |
| Complex6 | 0.695 | -34.9 | 1M17 | Epidermal Growth Factor Receptor (EGFR) tyrosine kinase domain |
| Complex6 | 3.873 | -28.3 | 4JT5 | Mammalian target of rapamycin (mTOR) |
| Complex6 | 5.146 | -51.6 | 8Q61 | Human Akt2 |
| Complex6 | 5.326 | 13.6 | 4OBE | GDP-bound Human KRas |
| Complex6 | 5.349 | -38.1 | 1JDH | β-catenin and HTCF-4 |
| Complex6 | 6.262 | 68.7 | 1HCK | Human cyclin-dependent kinase 2 |
| Complex6 | 7.828 | -22.6 | 3RCD | Kinase domain of human epidermal growth factor receptor 2 (HER2) |

Table S7. Molecular docking scores (kcal/mol) and binding free energy of **complex 7** to targets.

| Compound | Docking score kcal/mol | MM-GBSA  Kcal/mol | Target PDB ID | Target description |
| --- | --- | --- | --- | --- |
| Complex7 | -6.962 | -60.6 | 5HMH | HDM2/p53 interaction |
| Complex7 | -6.514 | -34.7 | 8Q61 | Human Akt2 |
| Complex7 | -6.003 | -44.8 | 8HOI | B-cell lymphoma 2 (BCL2) |
| Complex7 | -4.768 | -56.8 | 3L3M | Poly (ADP-ribose) polymerase (PARP-1) |
| Complex7 | -3.858 | -23.6 | 2GU8 | Akt/protein kinase B |
| Complex7 | -3.585 | -29.7 | 1JFF | Alpha-Beta-tubulin dimer |
| Complex7 | -2.930 | -27.5 | 3RCD | Kinase domain of human epidermal growth factor receptor 2 (HER2) |
| Complex7 | -2.567 | -21.2 | 4OBE | GDP-bound Human KRas |
| Complex7 | -2.076 | -18.0 | 3D0E | Human AKT2 |
| Complex7 | 1.387 | -34.8 | 4JT5 | Mammalian target of rapamycin (mTOR) |
| Complex7 | 2.868 | -53.5 | 1HCK | Human cyclin-dependent kinase 2 |
| Complex7 | 3.179 | -21.2 | 1M17 | Epidermal Growth Factor Receptor (EGFR) tyrosine kinase domain |
| Complex7 | 4.687 | -54.8 | 4XV2 | B-Raf Kinase |
| Complex7 | 6.393 | 31.1 | 1O6L | Activated Akt/protein kinase B |
| Complex7 | 6.959 | -25.5 | 3PP0 | Kinase domain of human epidermal growth factor receptor 2 (HER2) |
| Complex7 | 7.153 | -41.5 | 1JDH | β-catenin and HTCF-4 |
| Complex7 | 10.817 | -50.3 | 4WXX | DNA methyltransferase_1 |

Table S8. Molecular docking scores (kcal/mol) and binding free energy of **complex 8** to target’s.

| Compound | Docking score kcal/mol | MM-GBSA  Kcal/mol | Target PDB ID | Target description |
| --- | --- | --- | --- | --- |
| Complex8 | -14.138 | -66.3 | 5HMH | HDM2/p53 interaction |
| Complex8 | -13.947 | -65.5 | 4WXX | DNA methyltransferase_1 |
| Complex8 | -11.287 | -44.5 | 8HOI | B-cell lymphoma 2 (BCL2) |
| Complex8 | -11.095 | -73.4 | 3L3M | Poly(ADP-ribose) polymerase (PARP-1) |
| Complex8 | -10.701 | 46.6 | 1HCK | Human cyclin-dependent kinase 2 |
| Complex8 | -10.664 | -52.5 | 8Q61 | Human Akt2 |
| Complex8 | -10.531 | -55.9 | 4XV2 | B-Raf Kinase |
| Complex8 | -9.259 | -39.6 | 3PP0 | Kinase domain of human epidermal growth factor receptor 2 (HER2) |
| Complex8 | -9.084 | -30.7 | 3D0E | Human AKT2 |
| Complex8 | -8.999 | 8.4 | 1O6L | Activated Akt/protein kinase B |
| Complex8 | -8.752 | 70.3 | 4OBE | GDP-bound Human KRas |
| Complex8 | -8.540 | -30.6 | 4JT5 | Mammalian target of rapamycin (mTOR) |
| Complex8 | -8.420 | -26.7 | 2GU8 | Akt/protein kinase B |
| Complex8 | -8.103 | -36.8 | 1M17 | Epidermal Growth Factor Receptor (EGFR) tyrosine kinase domain |
| Complex8 | -7.202 | -41.0 | 1JDH | β-catenin and HTCF-4 |
| Complex8 | -6.669 | -47.6 | 1JFF | Alpha-Beta-tubulin dimer |
| Complex8 | -4.028 | -25.2 | 3RCD | Kinase domain of human epidermal growth factor receptor 2 (HER2) |

Table S9. Molecular docking scores (kcal/mol) and binding free energy of **complex 9** to targets.

| Compound | Docking score kcal/mol | MM-GBSA  Kcal/mol | Target PDB ID | Target description |
| --- | --- | --- | --- | --- |
| Complex9 | -16.230 | -65.9 | 4WXX | DNA methyltransferase_1 |
| Complex9 | -13.265 | -40.5 | 5HMH | HDM2/p53 interaction |
| Complex9 | -12.187 | -75.7 | 3L3M | Poly (ADP-ribose) polymerase (PARP-1) |
| Complex9 | -11.586 | 8.9 | 1HCK | Human cyclin-dependent kinase 2 |
| Complex9 | -11.234 | -82.4 | 8Q61 | Human Akt2 |
| Complex9 | -9.883 | -26.9 | 3D0E | Human AKT2 |
| Complex9 | -9.658 | -34.0 | 1JFF | Alpha-Beta-tubulin dimer |
| Complex9 | -9.118 | 27.0 | 1O6L | Activated Akt/protein kinase B |
| Complex9 | -8.893 | -32.6 | 1M17 | Epidermal Growth Factor Receptor (EGFR) tyrosine kinase domain |
| Complex9 | -8.818 | -56.7 | 8HOI | B-cell lymphoma 2 (BCL2) |
| Complex9 | -8.278 | -30.8 | 3PP0 | Kinase domain of human epidermal growth factor receptor 2 (HER2) |
| Complex9 | -7.805 | -42.1 | 3RCD | Kinase domain of human epidermal growth factor receptor 2 (HER2) |
| Complex9 | -7.617 | -35.8 | 2GU8 | Akt/protein kinase B |
| Complex9 | -6.701 | -24.6 | 4JT5 | Mammalian target of rapamycin (mTOR) |
| Complex9 | -6.673 | -30.2 | 1JDH | β-catenin and HTCF-4 |

Table S10. Molecular docking scores (kcal/mol) and binding free energy of **complex 10** to targets.

| Compound | Docking score kcal/mol | MM-GBSA  Kcal/mol | Target PDB ID | Target description |
| --- | --- | --- | --- | --- |
| Complex10 | -12.164 | -52.2 | 5HMH | HDM2/p53 interaction |
| Complex10 | -11.949 | -66.5 | 4WXX | DNA methyltransferase_1 |
| Complex10 | -11.772 | 33.2 | 1O6L | Activated Akt/protein kinase B |
| Complex10 | -11.166 | 20.7 | 1HCK | Human cyclin-dependent kinase 2 |
| Complex10 | -11.137 | 5.6 | 4OBE | GDP-bound Human KRas |
| Complex10 | -10.686 | -45.8 | 3L3M | Poly (ADP-ribose) polymerase (PARP-1) |
| Complex10 | -10.595 | -31.8 | 1JFF | Alpha-Beta-tubulin dimer |
| Complex10 | -9.467 | -43.3 | 8HOI | B-cell lymphoma 2 (BCL2) |
| Complex10 | -9.422 | -39.1 | 4JT5 | Mammalian target of rapamycin (mTOR) |
| Complex10 | -9.095 | -26.7 | 3D0E | Human AKT2 |
| Complex10 | -9.056 | -58.0 | 1JDH | β-catenin and HTCF-4 |
| Complex10 | -8.960 | -48.9 | 3PP0 | Kinase domain of human epidermal growth factor receptor 2 (HER2) |
| Complex10 | -8.077 | -33.6 | 3RCD | Kinase domain of human epidermal growth factor receptor 2 (HER2) |
| Complex10 | -7.797 | -28.5 | 1M17 | Epidermal Growth Factor Receptor (EGFR) tyrosine kinase domain |
| Complex10 | -6.757 | -30.0 | 8Q61 | Human Akt2 |
| Complex10 | -5.531 | -25.3 | 2GU8 | Akt/protein kinase B |

Table S11. Molecular docking scores (kcal/mol) and binding free energy of **complex 11** to targets.

| Compound | Docking score kcal/mol | MM-GBSA  Kcal/mol | Target PDB ID | Target description |
| --- | --- | --- | --- | --- |
| Complex11 | -12.059 | -44.4 | 4WXX | DNA methyltransferase_1 |
| Complex11 | -11.780 | -49.5 | 3L3M | Poly (ADP-ribose) polymerase (PARP-1) |
| Complex11 | -10.568 | -18.7 | 2GU8 | Akt/protein kinase B |
| Complex11 | -10.184 | -38.6 | 4JT5 | Mammalian target of rapamycin (mTOR) |
| Complex11 | -10.170 | -32.5 | 5HMH | HDM2/p53 interaction |
| Complex11 | -9.855 | 43.3 | 4OBE | GDP-bound Human KRas |
| Complex11 | -9.357 | -31.5 | 1M17 | Epidermal Growth Factor Receptor (EGFR) tyrosine kinase domain |
| Complex11 | -9.344 | 33.8 | 1HCK | Human cyclin-dependent kinase 2 |
| Complex11 | -9.119 | -26.0 | 3D0E | Human AKT2 |
| Complex11 | -8.750 | -40.2 | 8HOI | B-cell lymphoma 2 (BCL2) |
| Complex11 | -8.523 | -4.0 | 3PP0 | Kinase domain of human epidermal growth factor receptor 2 (HER2) |
| Complex11 | -8.037 | 48.2 | 1O6L | Activated Akt/protein kinase B |
| Complex11 | -7.620 | -62.2 | 1JFF | Alpha-Beta-tubulin dimer |
| Complex11 | -7.559 | -1.4 | 1JDH | β-catenin and HTCF-4 |
| Complex11 | -5.813 | -36.4 | 8Q61 | Human Akt2 |
| Complex11 | -4.222 | -39.6 | 3RCD | Kinase domain of human epidermal growth factor receptor 2 (HER2) |

Table S12. Molecular docking scores (kcal/mol) and binding free energy of **complex 12** to targets.

| Compound | Docking score kcal/mol | MM-GBSA  Kcal/mol | Target PDB ID | Target description |
| --- | --- | --- | --- | --- |
| Complex12 | -3.910 | -40.9 | 5HMH | HDM2/p53 interaction |
| Complex12 | 1.639 | 72.1 | 1O6L | Activated Akt/protein kinase B |
| Complex12 | 1.877 | 90.7 | 1HCK | Human cyclin-dependent kinase 2 |
| Complex12 | 2.107 | -31.0 | 8HOI | B-cell lymphoma 2 (BCL2) |
| Complex12 | 8.596 | -51.1 | 3D0E | Human AKT2 |
| Complex12 | 15.509 | -49.8 | 1M17 | Epidermal Growth Factor Receptor (EGFR) tyrosine kinase domain |
| Complex12 | 29.541 | -48.8 | 3RCD | Kinase domain of human epidermal growth factor receptor 2 (HER2) |

Table S13. Molecular docking scores (kcal/mol) and binding free energy of **complex13** to targets.

| Compound | Docking score kcal/mol | MM-GBSA  Kcal/mol | Target PDB ID | Target description |
| --- | --- | --- | --- | --- |
| Complex13 | -2.843 | -53.7 | 1JFF | Alpha-Beta-tubulin dimer |
| Complex13 | 1.480 | -54.2 | 8HOI | B-cell lymphoma 2 (BCL2) |
| Complex13 | 2.489 | 5.8 | 3D0E | Human AKT2 |
| Complex13 | 4.811 | -28.3 | 4JT5 | Mammalian target of rapamycin (mTOR) |
| Complex13 | 9.572 | -23.8 | 8Q61 | Human Akt2 |
| Complex13 | 10.151 | -50.8 | 5HMH | HDM2/p53 interaction |
| Complex13 | 16.208 | 46.5 | 4OBE | GDP-bound Human KRas |
| Complex13 | 20.987 | -71.1 | 3PP0 | Kinase domain of human epidermal growth factor receptor 2 (HER2) |

Table S14. Molecular docking scores (kcal/mol) and binding free energy of **complex 14** to targets.

| Compound | Docking score kcal/mol | MM-GBSA  Kcal/mol | Target PDB ID | Target description |
| --- | --- | --- | --- | --- |
| Complex14 | -2.935 | -42.3 | 8HOI | B-cell lymphoma 2 (BCL2) |
| Complex14 | -2.385 | -40.6 | 1M17 | Epidermal Growth Factor Receptor (EGFR) tyrosine kinase domain |
| Complex14 | -1.093 | -25.1 | 4WXX | DNA methyltransferase_1 |
| Complex14 | 1.748 | 33.0 | 1O6L | Activated Akt/protein kinase B |
| Complex14 | 2.799 | -14.3 | 4OBE | GDP-bound Human KRas |
| Complex14 | 3.805 | -53.0 | 1JFF | Alpha-Beta-tubulin dimer |
| Complex14 | 8.776 | -31.5 | 3L3M | Poly (ADP-ribose) polymerase (PARP-1) |
| Complex14 | 17.493 | -66.1 | 5HMH | HDM2/p53 interaction |
| Complex14 | 21.367 | 199.5 | 1HCK | Human cyclin-dependent kinase 2 |

Table S15. Molecular docking scores (kcal/mol) and binding free energy of **complex 15** to targets.

| Compound | Docking score kcal/mol | MM-GBSA  Kcal/mol | Target PDB ID | Target description |
| --- | --- | --- | --- | --- |
| Complex15 | -5.281 | -69.1 | 5HMH | HDM2/p53 interaction |
| Complex15 | -3.429 | -38.5 | 4JT5 | Mammalian target of rapamycin (mTOR) |
| Complex15 | -1.864 | -64.8 | 8HOI | B-cell lymphoma 2 (BCL2) |
| Complex15 | 1.094 | 92.8 | 4OBE | GDP-bound Human KRas |

Table S16. Molecular docking score (kcal/mol) and binding free energy of **complex 16** to targets.

| Compound | Docking score kcal/mol | MM-GBSA  Kcal/mol | Target PDB ID | Target description |
| --- | --- | --- | --- | --- |
| Complex16 | -5.547 | -46.0 | 5HMH | HDM2/p53 interaction |
| Complex16 | -3.912 | -50.7 | 8HOI | B-cell lymphoma 2 (BCL2) |
| Complex16 | -3.409 | -32.0 | 3D0E | Human AKT2 |
| Complex16 | 4.499 | 19.5 | 4OBE | GDP-bound Human KRas |
| Complex16 | 20.276 | -29.9 | 1M17 | Epidermal Growth Factor Receptor (EGFR) tyrosine kinase domain |

Table S17. Molecular docking score (kcal/mol) and binding free energy of **complex 17** to targets.

| Compound | Docking score kcal/mol | MM-GBSA  Kcal/mol | Target PDB ID | Target description |
| --- | --- | --- | --- | --- |
| Complex17 | -4.319 | -38.8 | 5HMH | HDM2/p53 interaction |
| Complex17 | -2.626 | -28.2 | 8HOI | B-cell lymphoma 2 (BCL2) |
| Complex17 | 0.112 | -54.1 | 1JFF | Alpha-Beta-tubulin dimer |
| Complex17 | 4.507 | -7.8 | 4JT5 | Mammalian target of rapamycin (mTOR) |
| Complex17 | 9.243 | -59.5 | 3RCD | Kinase domain of human epidermal growth factor receptor 2 (HER2) |

Table S18. Molecular docking scores (kcal/mol) and binding free energy of **complex 18** to targets.

| Compound | Docking score kcal/mol | MM-GBSA  Kcal/mol | Target PDB ID | Target description |
| --- | --- | --- | --- | --- |
| Complex18 | 2.973 | -35.5 | 3L3M | Poly(ADP-ribose) polymerase (PARP-1) |
| Complex18 | 3.553 | -16.8 | 1JFF | Alpha-Beta-tubulin dimer |
| Complex18 | 4.217 | 122.7 | 4OBE | GDP-bound Human KRas |
| Complex18 | 5.121 | -52.8 | 5HMH | HDM2/p53 interaction |
| Complex18 | 16.609 | -33.6 | 1M17 | Epidermal Growth Factor Receptor (EGFR) tyrosine kinase domain |

Table S19. Molecular docking scores (kcal/mol) and binding free energy of **complex 19** to targets.

| Compound | Docking score kcal/mol | MM-GBSA  Kcal/mol | Target PDB ID | Target description |
| --- | --- | --- | --- | --- |
| Complex19 | No binding pose was returned by Glide | | | |

Table S20. Molecular docking scores (kcal/mol) and binding free energy of **complex 20** to targets.

| Compound | Docking score kcal/mol | MM-GBSA  Kcal/mol | Target PDB ID | Target description |
| --- | --- | --- | --- | --- |
| Complex20 | -4.021 | -56.8 | 5HMH | HDM2/p53 interaction |
| Complex20 | -0.762 | 30.1 | 2GU8 | Akt/protein kinase B |
| Complex20 | 0.104 | 34.1 | 1O6L | Activated Akt/protein kinase B |
| Complex20 | 3.458 | -51.4 | 8HOI | B-cell lymphoma 2 (BCL2) |
| Complex20 | 7.856 | -42.8 | 1JFF | Alpha-Beta-tubulin dimer |
| Complex20 | 10.274 | -22.4 | 4WXX | DNA methyltransferase_1 |
| Complex20 | 13.848 | -29.6 | 4JT5 | Mammalian target of rapamycin (mTOR) |
| Complex20 | 16.223 | -25.2 | 3D0E | Human AKT2 |

Statistics analysis of the complexes

Table S21: Statistical analysis data table of complexes with the IC_50_, structure discription, MM-GBSA and docking score

| **Complex** | **IC50** | | | | **MM-GBSA bind** | | | | **Structure description** | | | | **Docking score** | | | |
| --- | --- | --- | --- | --- | --- | --- | --- | --- | --- | --- | --- | --- | --- | --- | --- | --- |
|  | **WI-38** | **MCF7** | **HCT116** | **PC3** | **4WXX** | **5HMH** | **3L3M** | **8Q61** | **Type** | **Acetylated** | **Chain length** | **Linker** | **4WXX** | **5HMH** | **3L3M** | **8Q61** |
| **Complex1** |  | **44.84** | **52.71** | **33.78** | **-69.9** | **-51.4** | **-52.8** | **-46** | **Mono** | **0** | **2** | **0** | **-11.641** | **-11.17** | **-9.348** | **-8.644** |
| **Complex2** | **50.50** | **15.10** | **5.71** | **6.88** | **-47.9** | **-56.8** | **-58.7** | **-29.5** | **Mono** | **0** | **3** | **0** | **-10.394** | **-9.89** | **-12.259** | **-8.613** |
| **Complex3** | **12.50** | **26.88** | **7.76** | **6.10** | **-52.4** | **-67.4** | **-55.8** | **-64.1** | **Mono** | **0** | **4** | **0** | **-11.177** | **-11.82** | **-7.45** | **-8.477** |
| **Complex4** | **12.00** | **29.75** | **36.80** | **20.30** | **-55.3** | **-65.1** | **-39.2** | **-41.4** | **Mono** | **0** | **5** | **0** | **-11.381** | **-9.891** | **-8.587** | **-6.818** |
| **Complex5** | **6.64** | **1.94** | **6.62** | **2.20** | **-38.7** | **-76** | **-59.5** | **-52.7** | **Mono** | **1** | **2** | **0** | **0.346** | **-7.229** | **-5.095** | **0.311** |
| **Complex6** | **2.35** | **2.30** | **3.80** | **2.21** | **-50.5** | **-58.1** | **-41.7** | **-51.6** | **Mono** | **1** | **3** | **0** | **-4.703** | **-7.944** | **0.326** | **5.146** |
| **Complex7** | **33.20** | **2.63** | **6.75** | **4.81** | **-50.3** | **-60.6** | **-56.8** | **-34.7** | **Mono** | **1** | **4** | **0** | **10.817** | **-6.962** | **-4.768** | **-6.514** |
| **Complex8** |  | **3.85** | **20.56** | **0.00** | **-65.5** | **-66.3** | **-73.4** | **-52.5** | **Di** | **0** | **2** | **2** | **-13.947** | **-14.138** | **-11.094** | **-10.664** |
| **Complex9** |  |  |  |  | **-65.9** | **-40.5** | **-75.7** | **-82.4** | **Di** | **0** | **3** | **2** | **-16.23** | **-13.265** | **-12.187** | **-11.233** |
| **Complex10** | **1.89** | **3.85** | **0.90** | **0.08** | **-66.5** | **-52.2** | **-45.8** | **-30** | **Di** | **0** | **4** | **2** | **-11.949** | **-12.164** | **-10.686** | **-6.757** |
| **Complex11** | **11.57** | **2.84** | **0.63** | **0.22** | **-44.4** | **-32.5** | **-49.5** | **-36.4** | **Di** | **0** | **5** | **2** | **-12.059** | **-10.17** | **-11.78** | **-5.813** |
| **Complex12** | **0.56** | **0.24** | **0.79** | **0.01** |  | **-40.9** |  |  | **Di** | **1** | **2** | **2** |  | **-3.91** |  |  |
| **Complex13** | **1.39** | **0.70** | **0.25** | **0.03** |  | **-50.8** |  | **-23.8** | **Di** | **1** | **2** | **3** |  | **10.151** |  | **9.572** |
| **Complex14** | **19.90** | **0.14** | **0.84** | **0.14** | **-25.1** | **-66.1** | **-31.5** |  | **Di** | **1** | **2** | **4** | **-1.093** | **17.493** | **8.776** |  |
| **Complex15** | **3.92** | **0.17** | **1.18** | **0.00** |  | **-69.1** |  |  | **Di** | **1** | **3** | **2** |  | **-5.281** |  |  |
| **Complex16** | **3.75** | **0.12** | **0.77** | **0.00** |  | **-46** |  |  | **Di** | **1** | **3** | **3** |  | **-5.547** |  |  |
| **Complex17** | **3.43** | **2.28** | **0.27** | **0.03** |  | **-38.8** |  |  | **Di** | **1** | **3** | **4** |  | **-4.319** |  |  |
| **Complex18** | **1.51** | **1.81** | **0.39** | **0.04** |  | **-52.8** | **-35.5** |  | **Di** | **1** | **4** | **2** |  | **5.121** | **2.973** |  |
| **Complex19** | **1.29** | **2.16** | **0.28** | **0.03** |  |  |  |  | **Di** | **1** | **4** | **3** |  |  |  |  |
| **Complex20** | **3.63** | **0.97** | **0.74** | **0.56** | **-22.4** | **-56.8** |  |  | **Di** | **1** | **4** | **4** | **10.274** | **-4.021** |  |  |

Table S22: Correlation results of complex type verse docking score.


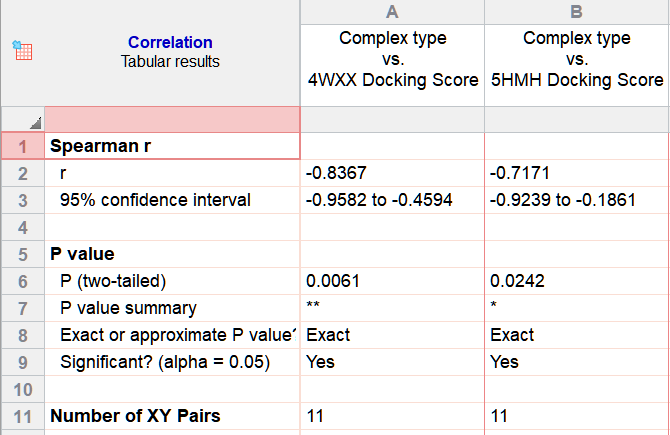


Table S23: Correlation results of docking score verse verse MCF IC50 and acetylation.


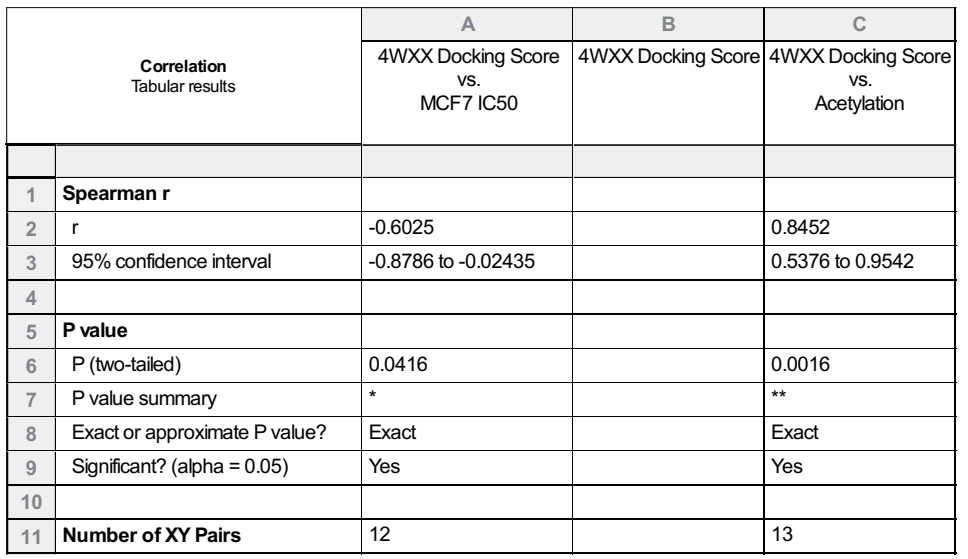


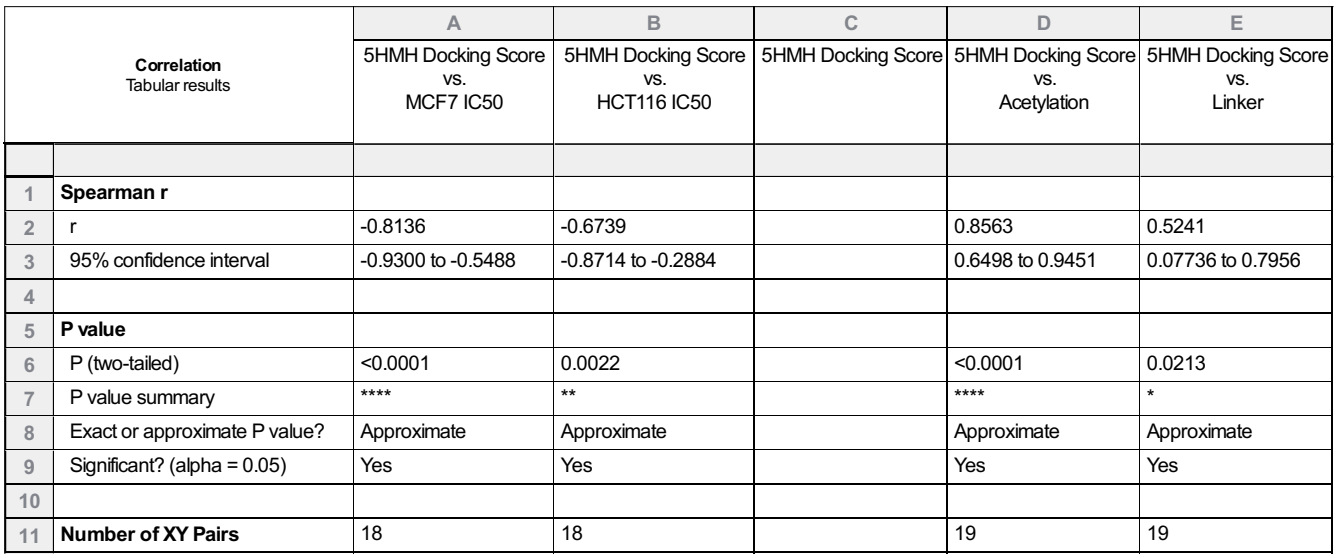


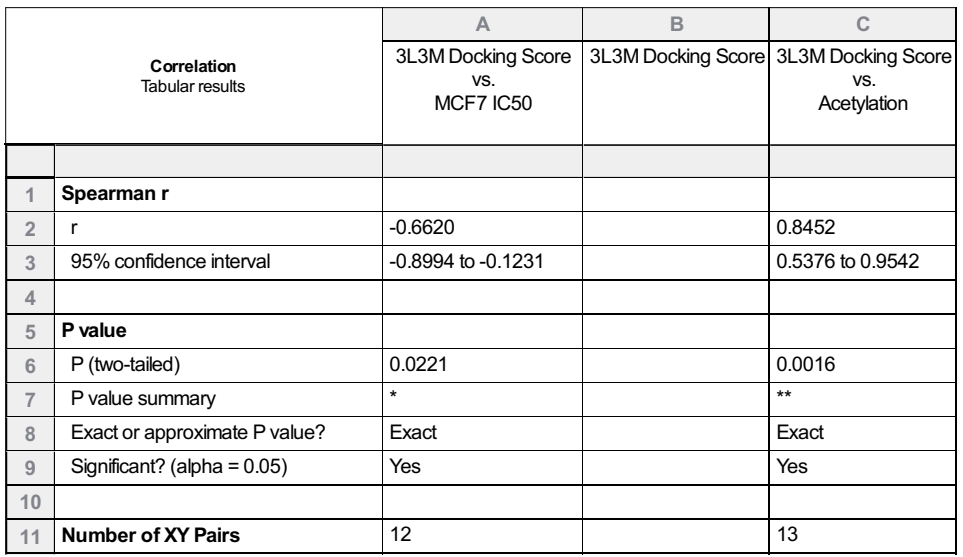


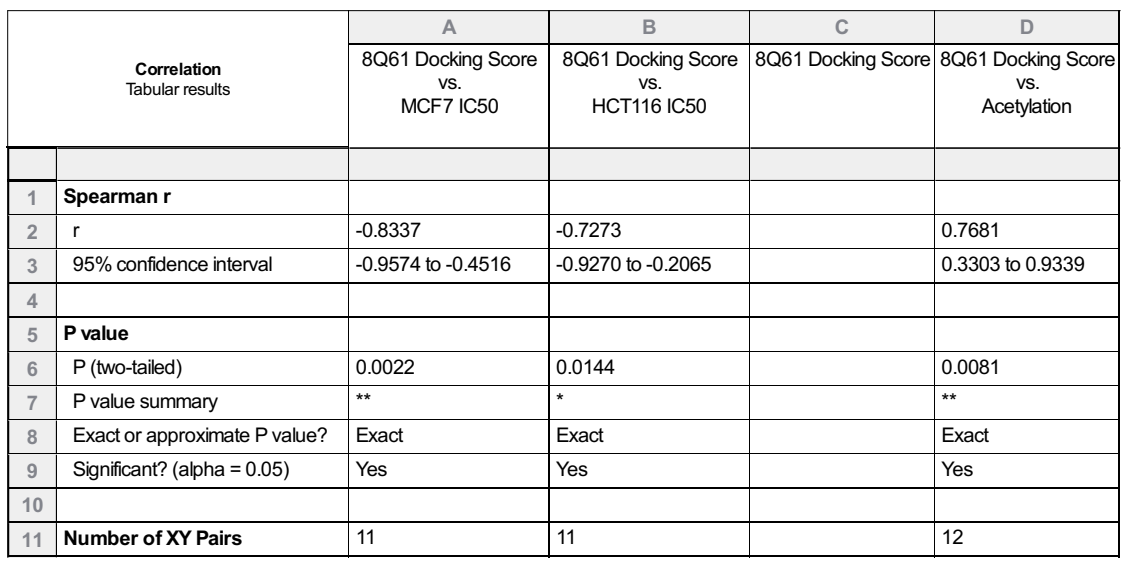


**Figures:**

Figure S1. The binding free energies (Kcal/mol) for the various complexes with Human cyclin-dependent kinase-2 (1HCK) target

Figure S2: The binding free energies (Kcal/mol) for the various complexes with Activated Akt/protein kinase-B (1O6L) target

Figure S3. The binding free energies (Kcal/mol) for the various complexes with Akt/Protein kinase B (2GUB) target

Figure S4. The binding free energies (Kcal/mol) for the various complexes with Human Akt2 (3D0E) target

Figure S5. The binding free energies (Kcal/mol) for the various complexes with Poly(ADP-ribose) polymerase (PARP1) (3L3M) target

Figure S6. The binding free energies (Kcal/mol) for the various complexes with (HER2) Human epidermal growth factor receptor-2 (3PP0) target

Figure S7. The binding free energies (Kcal/mol) for the various complexes with (HER2) Human epidermal growth factor receptor-2 (3RCD) target

Figure S8. The binding free energies (Kcal/mol) for the various complexes with Mammalian target of rapamycin (mTOR)(4JT5) target

Figure S9. The binding free energies (Kcal/mol) for the various complexes with GDP-bound Human KRas (4OBE) target

Figure S10. The binding free energies (Kcal/mol) for the various complexes with DNA methyltransferase-1 (4WXX) target

Figure S11. The binding free energies (Kcal/mol) for the various complexes with B-Raf Kinase (4XV2) target

Figure S12. The binding free energies (Kcal/mol) for the various complexes with HDM2/ P53 interaction (5HMH) target

Figure S13. The binding free energies (Kcal/mol) for the various complexes with B-cell lymphoma 2 (BCL2) (8HOI) target

Figure S14. The binding free energies (Kcal/mol) for the various complexes with Epidermal Growth Factor Receptor (1M17) target

1. Corresponding Author. Email: cadokoh@ucc.edu.gh [↑](#footnote-ref-1)
